# Securing diagonal integration of multimodal single-cell data against ambiguous mapping

**DOI:** 10.1093/bioinformatics/btaf345

**Published:** 2025-06-14

**Authors:** Han Zhou, Kai Cao, Yang Young Lu

**Affiliations:** Cheriton School of Computer Science, University of Waterloo, Waterloo, ON, N2L 3G1, Canada; Eric and Wendy Schmidt Center, Broad Institute of MIT and Harvard, Boston, MA, 02142, United States; Cheriton School of Computer Science, University of Waterloo, Waterloo, ON, N2L 3G1, Canada

## Abstract

**Motivation:**

Recent advances in single-cell multimodal omics technologies enable the exploration of cellular systems at unprecedented resolution, leading to the rapid generation of multimodal datasets that require sophisticated integration methods. Diagonal integration has emerged as a flexible solution for integrating heterogeneous single-cell data without relying on shared cells or features. However, the absence of anchoring elements introduces the risk of artificial integrations, where cells across modalities are incorrectly aligned due to ambiguous mapping.

**Results:**

To address this challenge, we propose SONATA (Securing diagOnal iNtegrATion against Ambiguous) mapping, a novel diagnostic method designed to detect potential artificial integrations resulting from ambiguous mappings in diagonal data integration. SONATA identifies ambiguous alignments by quantifying cell–cell ambiguity within the data manifold, ensuring that biologically meaningful integrations are distinguished from spurious ones. It is worth noting that SONATA is not designed to replace any existing pipelines for diagonal data integration; instead, SONATA works simply as an add-on to an existing pipeline for achieving more reliable integration. Through a comprehensive evaluation on both simulated and real multimodal single-cell datasets, we observe that artificial integrations in diagonal data integration are widespread yet surprisingly overlooked, occurring across all mainstream diagonal integration methods. We demonstrate SONATA’s ability to safeguard against misleading integrations and provide actionable insights into potential integration failures across mainstream methods. Our approach offers a robust framework for ensuring the reliability and interpretability of multimodal single-cell data integration.

**Availability and implementation:**

The source code is available at (https://github.com/batmen-lab/SONATA).

## 1 Introduction

Recent advances in single-cell multimodal omics technologies now allow biologists to simultaneously capture unprecedented high-resolution omics profiles at the cellular level (Vandereyken *et al.* 2023). Numerous technologies have been developed to explore cellular systems from diverse perspectives, resulting in multiple data modalities, each capturing a distinct molecular aspect of the cell. These modalities include scRNA-seq for gene expression (Tang *et al.* 2009), scATAC-seq for chromatin accessibility (Buenrostro *et al.* 2015), scMethyl-seq for DNA methylation (Smallwood *et al.* 2014), scHi-C for chromatin 3D conformation (Nagano *et al.* 2013), and others. Consequently, the integrative analysis of vast single-cell multimodal omics data has garnered increasing interest, with the potential to provide deeper insights into cellular processes that were previously inaccessible through studies of bulk cells.

Despite its great potential, integrating single-cell data modalities for downstream interpretation remains a complex challenge (Argelaguet *et al.* 2021). First of all, different data modalities may not share the same set of features. For example, scRNA-seq focuses on gene expression, while scATAC-seq measures chromatin accessibility across various genomic regions. Beyond feature discrepancies, another challenge arises from the destructive nature of profiling technologies, which results in different data modalities measuring distinct sets of cells. For example, both scATAC-seq and scHi-C target genomic DNA, meaning that each individual cell can only be measured once due to genome cleavage. Although recent technological advances allow for joint profiling of some modalities in the same cells (such as scRNA-seq and scATAC-seq), many modalities still cannot be measured together. Additionally, many single-cell datasets have already been collected over the years using distinct sets of cells. Therefore, maximizing the utility of these datasets through integration is essential, rather than disregarding valuable data collected with distinct cell sets. Lastly, intrinsic variations exist between datasets, even within the same modality. These variations can be biological, arising from samples collected under different conditions across tissues, organs, individuals, or species. They can also be technical, resulting from differences in laboratory instruments and experimental protocols used during sample profiling. In summary, the absence of shared cells or features across single-cell modalities makes data integration a highly challenging task.

**Figure 1.**
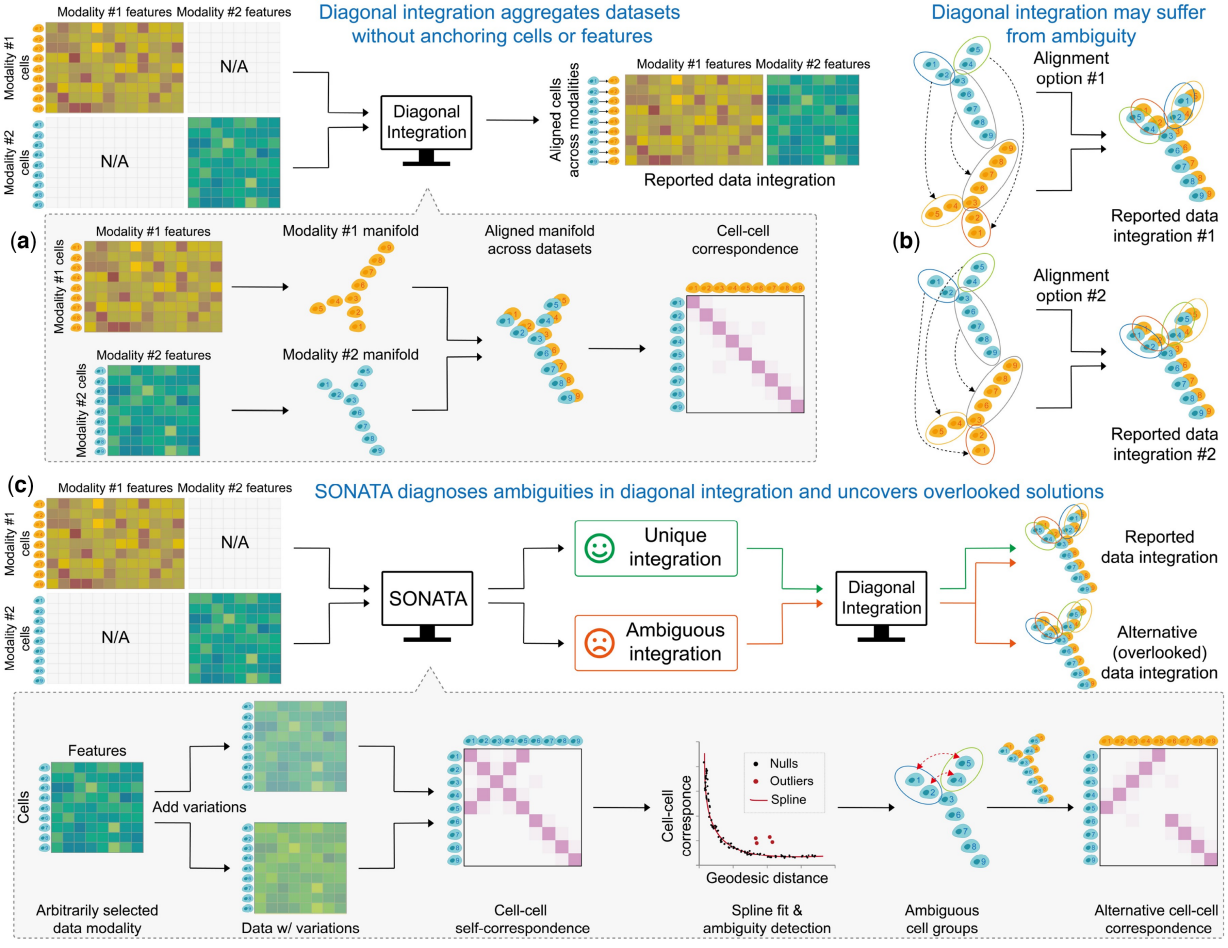
Overview of SONATA. (a) SONATA is designed to safeguard diagonal integration of multimodal single-cell data. (b) SONATA identifies potential artificial integrations arising from ambiguous mapping, which occurs when two cells that are far apart in the data manifold share similar geometric contexts. (c) SONATA uses a novel cell–cell ambiguity measurement to provide insights into possible alternative integration solutions that are often overlooked by existing integration methods. The ambiguity is measured using a statistical confidence measure for each pair of cells within the same modality. This ambiguity is then used to identify cell groups with similar geometric contexts, which could potentially lead to artificial integrations in cross-modality mapping.

In recent years, a growing number of integration methods have been developed to tackle multimodal single-cell data integration, using different approaches depending on the availability of shared features or shared cells as anchors (Liu *et al.* 2024). Among these methods, the “diagonal integration” approach—the focus of this paper—has garnered increasing attention, as it does not rely on anchoring cells or features for integration, making it the most flexible option with minimal prior knowledge. In contrast, methods that rely on anchored features or cells are only applicable when it is possible to engineer matched features or when multiple modalities can be jointly profiled within the same cell. From a practitioner’s perspective, an effective diagonal integration method would significantly broaden the scope of potential data integration, making it highly appealing to the community. However, the absence of anchors also introduces greater challenges in achieving accurate integration results (Xu and McCord 2022). Grounded in empirical evidence suggesting that each data modality occupies a low-dimensional manifold with latent semantic structure (Welch *et al.* 2017), we note that diagonal integration methods, including MMD-MA (Liu *et al.* 2019), SCIM (Stark *et al.* 2020), SCOT (Demetci *et al.* 2022a, b), UnionCom (Cao *et al.* 2020), and Pamona (Cao *et al.* 2021), typically involve three steps (Fig. 1a): (i) Projecting different modalities into a shared space to reveal their underlying manifold structures, (ii) Identifying correspondence relationships between cells across data modalities, and (iii) Generating an integrated profile from all modalities. Although the methods differ in implementation details, they all share the same principle of aligning data within a low-dimensional manifold (Xu and McCord 2022).

Despite the varied implementations of manifold alignment, all existing diagonal integration methods share an implicit yet critical assumption: data from different modalities are generated from a similar distribution, as maintained by the shared manifold (Xu and McCord 2022). As a consequence, these methods indiscriminately provide an integration solution along with corresponding cross-modality correspondence relationships. As a user, a natural question arises: “how can one distinguish between true biological integration—accurately matching cells across different modalities—and any potential artificial integration?” Specifically, an artificial integration solution can be mathematically indistinguishable from the accurate biological solution we seek. Studies have reported that multiple alignments can appear equally optimal, potentially leading to artificial alignments (Yang *et al.* 2021). As a motivating example, consider the cell cycle (Schafer 1998), a periodic biological process in human cells. The periodicity of cycling cells in different transcriptomic states forms a circular trajectory, with a cell’s position on the trajectory indicating its timing in the cycle. In this case, an infinite number of incorrect mappings can exist between two cycles, as rotating one cycle by arbitrary degrees naturally produces new, albeit incorrect, solutions. Without a systematic mechanism to flag potential artificial integrations, blindly integrating single-cell datasets can lead to unfaithful and misleading interpretations (Chari and Pachter 2023).

In this paper, we propose a novel diagnostic method for diagonal data integration of multimodal single-cell data, Securing diagOnal iNtegrATion against Ambiguous (SONATA) mapping, that aims to identify potential artificial integrations resulting from ambiguous mapping (Fig. 1). Here, ambiguous mapping does not refer to substituting one cell with another within the same modality due to their similarity in features or close proximity in the underlying manifold. Instead, it occurs when two cells that are far apart in the data manifold share similar geometric contexts, leading to potential confusion during integration. For example, as illustrated in Fig. 1b, cell 1 in data modality 1 is expected to map to cell 1 in data modality 2. Ambiguous mapping refers to the scenario where cell 1 in data modality 1 is instead mapped to cell 5 in data modality 2. It’s important to note that mapping cell 1 in data modality 1 to cell 2 in data modality 2 is not considered ambiguous; this could simply result from variations in the data or the inherent instability of diagonal integration methods. Unlike existing methods that recklessly report an integration solution, SONATA distinguishes whether the integration is unique or if alternative solutions exist, prompting users to carefully discern the true biological solution before proceeding to downstream analysis.

The novelty of SONATA is 2-fold. First and foremost, we demonstrate that artificial integrations resulting from ambiguous mapping in diagonal data integration are widespread yet surprisingly overlooked, occurring across all mainstream diagonal integration methods. Note that artificial integrations are more harmful than failed integrations because, while failed integrations can be qualitatively recognized, artificial integrations are difficult to detect and can mislead users into pursuing hypotheses based on erroneous results. For this reason, we propose a systematic strategy to detect potential artificial integrations without relying on prior knowledge (Fig. 1c). The core idea is centered on a novel cell–cell ambiguity measurement, where two cells are considered ambiguous—and thus likely to substitute for each other in a cross-modality alignment—given that they are far apart in the data manifold. By aligning a data modality to a variational version of itself, ambiguity is assessed through a statistical confidence measure for each pair of cells within the same modality. This is based on their observed correspondences relative to a null model, in which the correspondence probability diminishes as the cell–cell distance increases along the data manifold. Consequently, the cell–cell ambiguities provide insights into possible alternative integration solutions that are often overlooked by existing integration methods.

We applied SONATA to both simulated and real multimodal single-cell datasets to demonstrate its empirical utility. Our experiments on real datasets highlight SONATA’s ability to safeguard diagonal integration of gene expression, DNA methylation, and chromatin accessibility modalities against ambiguous mapping from mainstream methods, effectively informing potential users where these methods might fail. It is worth noting that SONATA is not designed to replace any existing pipelines for diagonal data integration; instead, SONATA works simply as an add-on to an existing pipeline for achieving more reliable integration. In summary, SONATA’s diagnostic analysis is a pioneering effort that offers guidelines for the reliable use of diagonal integration methods, ensuring robust integration of single-cell data.

## 2 Materials and methods

### 2.1 Approach

#### 2.1.1 Problem setup

Let the two datasets of cells be $X={\left\{x_{i}\right\}}_{i=1}^{n_{x}}\in R^{n_{x}\times p_{x}}$ in data modality X and $Y={\left\{y_{j}\right\}}_{j=1}^{n_{y}}\in R^{n_{y}\times p_{y}}$ in data modality Y, respectively. The numbers of cells in the two data modalities are $n_{x}$ and $n_{y}$, and the feature dimensions are $p_{x}$ and $p_{y}$, respectively. Given *X* and *Y* as input, existing diagonal integration methods uncover a low-dimensional manifold that captures the covariation between *X* and *Y* and identify correspondence relationships between cells across the modalities (Fig. 1a). Each data modality is typically represented as a low-dimensional manifold by constructing a geodesic distance matrix between the cells. To achieve this, a weighted *k*-nearest neighbor (*k*-NN) graph of cells within each data modality is constructed (Costa and Hero 2004). Subsequently, the shortest distance between each node pair on the graph is calculated, as these shortest distances serve as approximations of geodesic distances on the data manifold (Tenenbaum *et al.* 2000). The resultant geodesic matrices for *X* and *Y* are denoted as $K_{x}\in R^{n_{x}\times n_{x}}$ and $K_{y}\in R^{n_{y}\times n_{y}}$, respectively.

Once two datasets are aligned, the identified correspondence relationships are encoded into a cross-modality correspondence matrix $\Gamma_{xy}\in R^{n_{x}\times n_{y}}$, which is generated either explicitly (Cao *et al.* 2020, Demetci *et al.* 2022a) or implicitly (Liu *et al.* 2019, Stark *et al.* 2020), where ${\left[\Gamma_{xy}\right]}_{ij}$ represents the probability of the *i*-th cell in X aligning with the *j*-th cell in Y. SONATA focuses on diagnosing diagonal data integration by assessing whether the resulting integration may be artificial. If so, SONATA is designed to enumerate all possible solutions that may have been overlooked, ensuring that the true biological solution is included among the possibilities.

#### 2.1.2 Revealing the manifold ambiguity between cells

SONATA accepts any arbitrarily selected data modality as input and identifies ambiguous cells that could potentially result in artificial integrations during cross-modality mapping. Without loss of generality, we designate *X* as the selected data modality with the corresponding geodesic matrix $K_{x}\in R^{n_{x}\times n_{x}}$. We reason that if such ambiguity exists, it indicates that certain regions of the data manifold resemble others, implying that these regions should align when the data is mapped against itself. However, utilizing self-alignment of the given data modality for ambiguity detection is challenging, as a trivial solution always exists where each cell aligns with itself rather than with geometrically similar cells that are far apart in the data manifold. To prevent a trivial solution, we repeatedly align the data modality to different variational versions of itself by introducing random Gaussian noise and varying the number of neighbors in manifold construction (Fig. 1c). (Refer to Section 5, available as supplementary data at *Bioinformatics* online for the construction details). We denote the variational data matrix and its corresponding geodesic matrix as $\tilde{X}={\left\{{\tilde{x}}_{j}\right\}}_{j=1}^{n_{x}}$ and ${\tilde{K}}_{x}\in R^{n_{x}\times n_{x}}$, respectively.

With the variations of the data modality, we perform self-alignment using the Gromov–Wasserstein optimal transport (Mémoli 2011, Peyré and Cuturi 2019) optimal transport (Mémoli 2011, Peyré and Cuturi 2019), disregarding the self-correspondence between cells. The rationale for using optimal transport for self-alignment lies in its ability to explicitly provide probabilistic cell–cell correspondences, facilitating the subsequent identification of ambiguous cells. Specifically, we first define a cost function L:R×R→R, which quantifies how transporting a pair of samples $x_{i}$ and $x_{k}$ in the selected data *X* onto another pair of samples ${\tilde{x}}_{j}$ and ${\tilde{x}}_{l}$ in the variational data $\tilde{X}$. A straightforward choice for the cost function is $L(x,\tilde{x})=\frac{1}{2}{\left(x-\tilde{x}\right)}^{2}$. Given the cost function, we further compute a fourth-order tensor $L\in R^{n_{x}\times n_{x}\times n_{y}\times n_{y}}$, where $L_{\mathrm{ijkl}}=L({\left[K_{x}\right]}_{ik},{\left[{\tilde{K}}_{x}\right]}_{jl})$. The cell–cell correspondences from self-alignment $\Gamma_{xx}\in R^{n_{x}\times n_{x}}$ can be obtained by solving:


(1) $$\underset{\Gamma_{xx}\in \Pi (p_{x},p_{\tilde{x}})}{\min}\langle L(K_{x},{\tilde{K}}_{x})⊗\Gamma_{xx},\Gamma_{xx}\rangle$$

where $p_{x},p_{\tilde{x}}\in R^{n_{x}}$ are the marginal distribution in *X* and $\tilde{X}$, respectively. And $\Pi (p_{x},p_{\tilde{x}})$ is the set of coupling matrices defined as: $\Pi (p_{x},p_{\tilde{x}})=\left\{\Gamma_{xx}\in R_{+}^{n_{x}\times n_{x}}:\Gamma_{xx}1_{n_{x}}=p_{x},\;\Gamma_{xx}^{T}1_{n_{x}}=p_{\tilde{x}}\right\}$. (Refer to Section 2, available as supplementary data at *Bioinformatics* online for the details of the Gromov–Wasserstein optimal transport explanation and optimization).

#### 2.1.3 Quantifying the revealed manifold ambiguity

The resulting cell–cell correspondences from self-alignment are used to evaluate manifold ambiguity between cells through a statistical confidence measure. Specifically, SONATA models an empirical null from the observed cell–cell correspondences, by observing how it diminishes conditioned on the geodesic distance between cells along the data manifold. To achieve this, SONATA fits a cubic smoothing spline to model the probability of cell–cell correspondence as a function of geodesic distance, with an anti-tonic regression constraint to ensure the probability is monotonically non-increasing as the distance increases (Fig. 1c).


(2) $$\begin{array}{c} \underset{\theta }{\min}{\left\|\Gamma_{xx}-f_{\theta }(K_{x})\right\|}_{F}^{2}\\ s.t.\;\;\forall \;{\left[K_{x}\right]}_{ij}>{\left[K_{x}\right]}_{kl}\Rightarrow f_{\theta }({\left[K_{x}\right]}_{ij})\leq f_{\theta }({\left[K_{x}\right]}_{kl}) \end{array}$$

where f:R→R is a 1D cubic smoothing spline parameterized by θ and ${\left\|\cdot \right\|}_{F}^{2}$ denotes the Frobenius norm, commonly known as the sum of squared error. Equation (2) can be optimized using the “LinearGAM” function from the pyGAM library (Serven and Brummitt 2018). (Refer to Section 4.1, available as supplementary data at *Bioinformatics* online for the details to facilitate smooth and efficient spline fit). Once the spline is fitted, we calculate the deviation between the observed and expected correspondence probabilities for each cell pair. A one-sided *P*-value is then computed for each deviation using a normality test to evaluate its statistical significance, with a cutoff threshold set at 1%. A statistically significant ambiguous cell pair indicates that these two cells share similar geometric contexts, potentially leading to artificial integrations in cross-modality mapping.

The presence of ambiguous cell pairs indicates the existence of alternative, yet overlooked, integration solutions–suggesting that the results produced by current diagonal integration methods may be artificial. Based on the intuition that cells engage in ambiguous alignments alongside their neighboring cells rather than individually, we apply a data-driven approach to aggregate ambiguous cells into more coherent and interpretable groups. Specifically, the aggregation of ambiguous cell pairs is framed as a constrained clustering problem (Basu *et al.* 2004), extending standard clustering by incorporating pairwise ambiguity constraints, which ensure that two cells identified as ambiguous cannot be placed within the same group.

The final number of ambiguous groups is selected using the Elbow method, where the number of violated constraints is plotted against the number of groups. As the number of groups increases, the constraint violations decrease, and the optimal group number is determined at the point where this decrease slows and begins to plateau. The rationale for these ambiguous cell groups, denoted as $G=\left\{G_{i}\subseteq \left\{1,2,\ldots ,n_{x}\right\}:i=1,2,\ldots \right\}$, is that one group can be substituted for another based on their geometric resemblance during cross-modality integration.

#### 2.1.4 Diagnosing diagonal integration solutions

Lastly, SONATA generates alternative integration solutions based on identified ambiguous cell groups (Fig. 1c). Specifically, for two ambiguous group of cells in G, denoted as $G_{s},G_{t}\in G$, we obtain their cell mapping $\Gamma_{st}={\left[\gamma_{ij}\right]}_{i,j=1}^{\left|G_{s}|,|G_{t}\right|}\in R^{\left|G_{s}|\times |G_{t}\right|}$ from the self-alignment conducted in Section. Revealing the manifold ambiguity between cells. Thereby, given an existing diagonal integration solution in the form of cross-modality correspondence matrix $\Gamma_{xy}\in R^{n_{x}\times n_{y}}$, SONATA reports an alternative solution $\Gamma_{xy}^{\text{alt}}=P_{st}\Gamma_{xy}\in R^{n_{x}\times n_{y}}$ where the permutation matrix $P_{st}={\left[P_{ij}\right]}_{i,j=1}^{n_{x}}\in R^{n_{x}\times n_{x}}$ is defined as:


(3) $$\begin{array}{c} P_{ij}=\{\begin{array}{cc} \gamma_{ij}, & i\in G_{s},j\in G_{t}\\ \gamma_{ji}, & j\in G_{s},i\in G_{t}\\ 1, & i,j\notin G_{s}\cup G_{t},i=j\\ 0, & \text{otherwise} \end{array} \end{array}$$

It is important to note that the alternative integration solutions identified are based on geometric resemblance rather than biological validity. However, revealing these possibilities encourages users to critically evaluate and discern the true biological solution, rather than relying on a potentially misleading outcome.

### 2.2 Datasets

**Figure 2.**
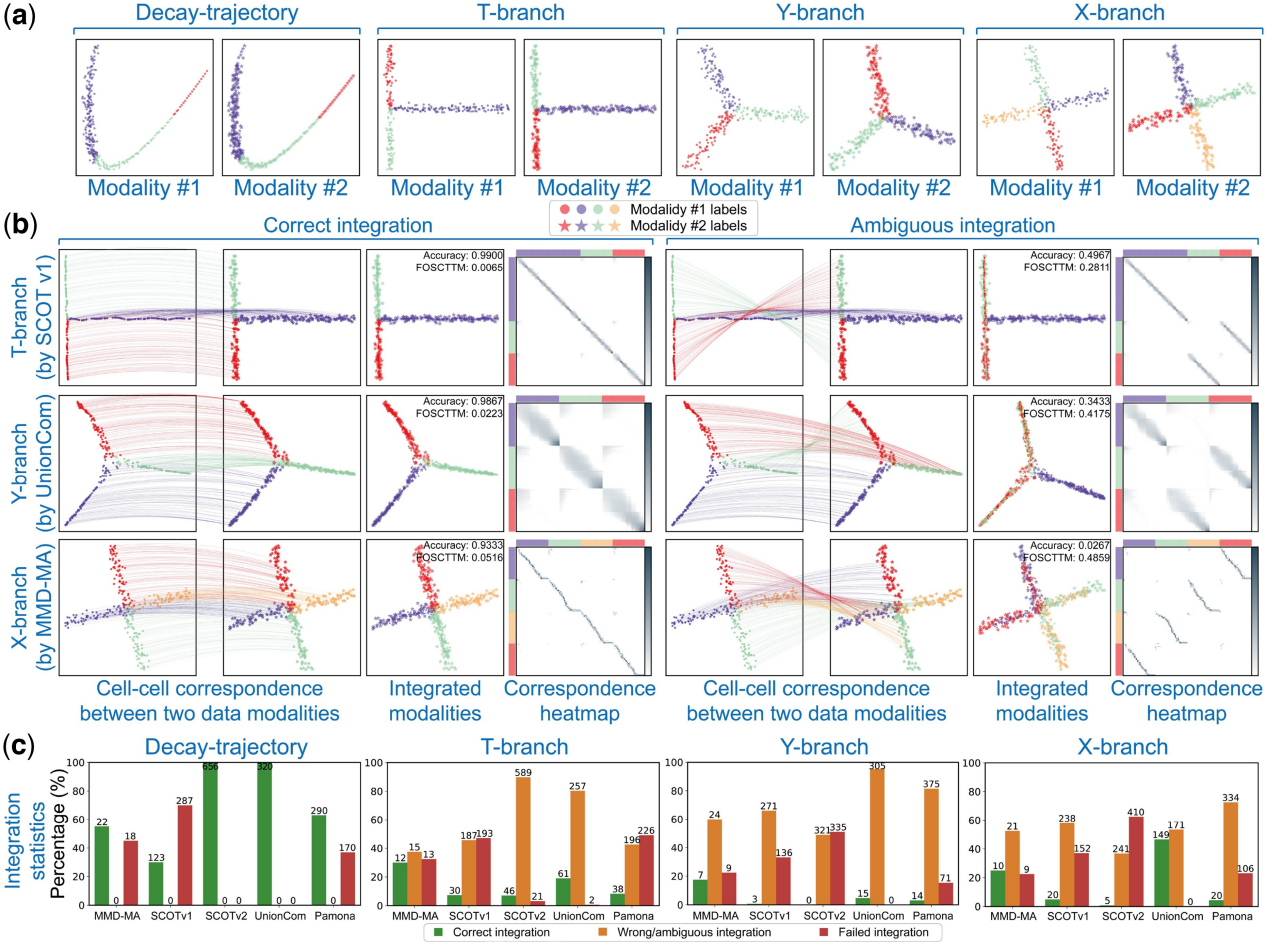
Ambiguous integrations are revealed on simulated datasets. (a) Four simulated datasets were constructed, with one dataset (i.e. Decay-path) having no ambiguity, while the other three exhibit branch-level ambiguity. The cell labels are indicated by color, where branches of the same color between two modalities should be aligned. Different modalities are represented by different markers. (b) Ambiguous mappings can be qualitatively detected by examining the cross-modality cell–cell correspondence matrix generated by existing integration methods, which forms the basis of SONATA’s intuition. (c) Ambiguous mappings occur universally across mainstream diagonal integration methods.

To uncover the artificial integration issues caused by ambiguous mapping, we first constructed four simulated datasets (Fig. 2a). The simulated datasets fall into two categories: one without ambiguity and three with ambiguity. The ambiguous datasets consist of T-shaped, Y-shaped, and X-shaped branches, designed to mimic bifurcating or multifurcating differentiation processes. The distinction among these datasets lies in the resemblance of two, three, and four branches, respectively. In contrast, the unambiguous dataset features a hook-like shape with a decaying density from one end to the other, where two manifolds are uniquely aligned without any ambiguity. Each simulated dataset comprises two modalities, each containing $n_{x}=n_{y}=300$ cells, with cell–cell correspondence information generated. The cells reside on a 2D manifold for both modalities, which are then mapped to feature spaces of $p_{x}=1000$ and $p_{y}=2000$ dimensions, respectively, with Gaussian noise (σ=0.1) added to the features. Given the differing feature dimensions between the modalities, no feature-level correspondence exists between them.

**Figure 3.**
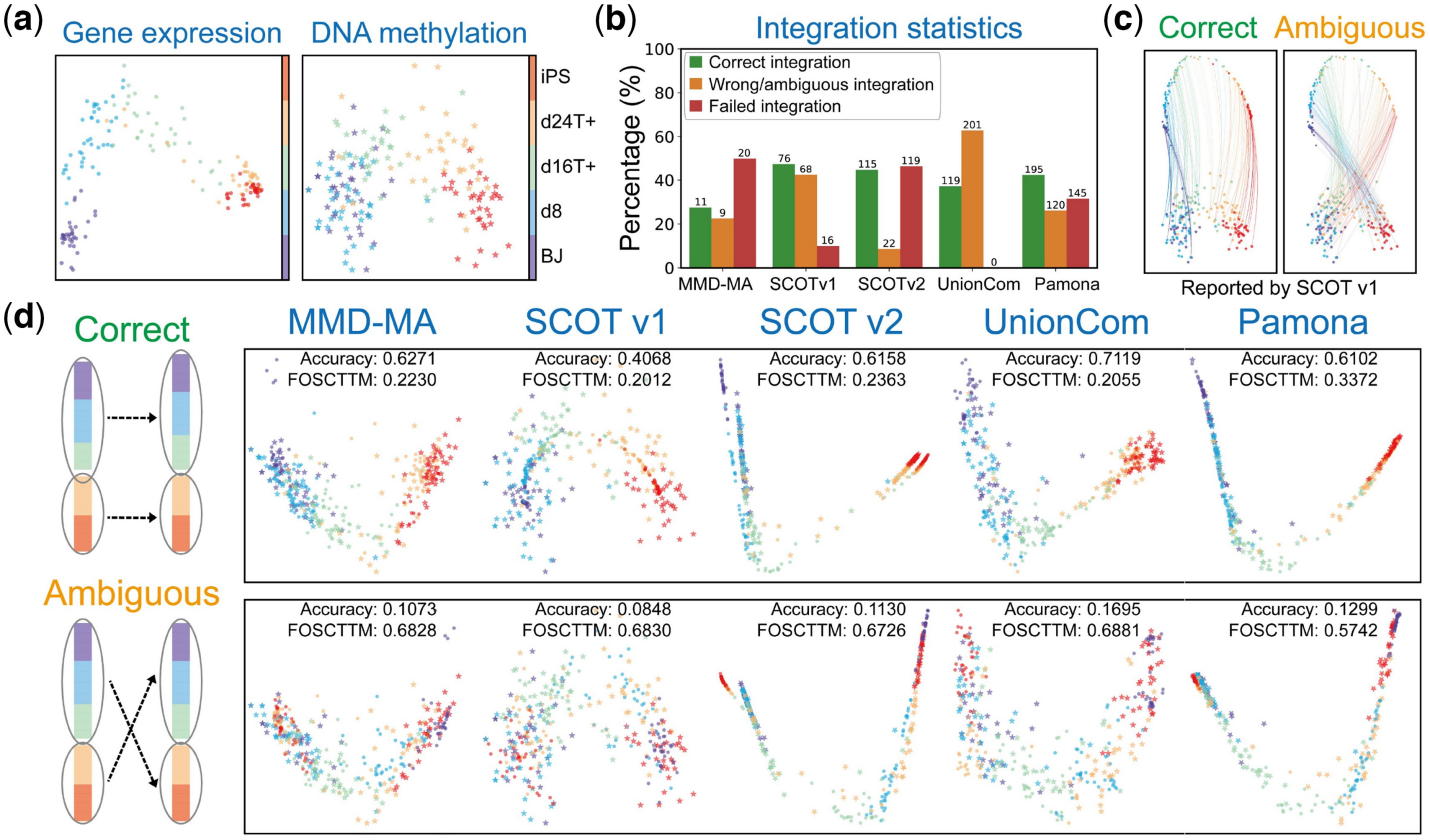
Ambiguous integrations are revealed on the sc-GEM dataset. (a) The sc-GEM dataset consists of two data modalities: gene expression and DNA methylation. (b) Ambiguous mappings occur universally across mainstream diagonal integration methods. It is important to note that ambiguous mappings can only be identified when ground truth labels are available, which are typically absent in real-world data. (c) Ambiguous mappings occur when one modality is reversely aligned to the other during the integration process. (d) Ambiguous mappings can be qualitatively revealed through the aligned manifold and the cross-modality cell–cell correspondences.

Alongside the simulated datasets, we used three real multimodal single-cell datasets, each with co-assayed cell–cell correspondence information, to uncover the artificial integration issues. The sc-GEM dataset (Fig. 3a) was generated using the sc-GEM technology (Cheow *et al.* 2016), a sequencing technology that simultaneously profiles gene expression and DNA methylation states within the same cell. The dataset was derived from human somatic cell samples undergoing conversion to induced pluripotent stem (iPS) cells (Cheow *et al.* 2016), which contains $n_{x}=n_{y}=177$ cells with $p_{x}=34$ dimensions of gene expression features and $p_{y}=27$ dimensions of chromatin accessibility features, respectively.

**Figure 4.**
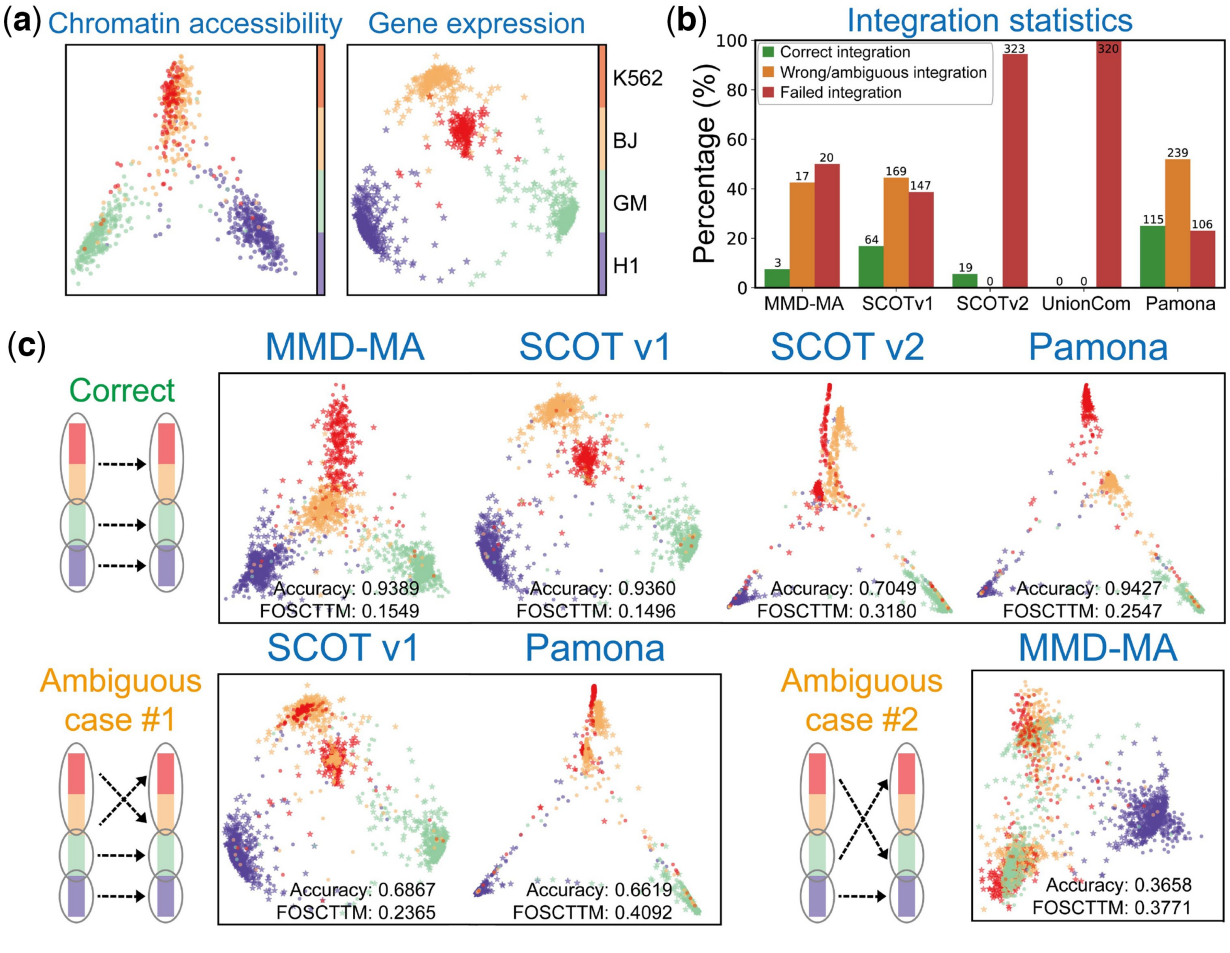
Ambiguous integrations are revealed on the SNARE-Seq dataset. (a) The dataset consists of two data modalities: chromatin accessibility and gene expression. (b) Ambiguous mappings occur across most diagonal integration methods, except for UnionCom, which always fails regardless of parameter settings. It is important to note that ambiguous mappings can only be identified when ground truth labels are available, which are typically absent in real-world data. (c) Correct integration accurately aligns cells of the same type across modalities. Artificial integrations can result from different types of ambiguous mappings.

The SNARE-Seq dataset (Fig. 4a) was generated using the SNARE-seq technology (Chen *et al.* 2019), a sequencing technology that simultaneously profiles gene expression and chromatin accessibility within the same cell. The dataset was derived from a mixture of BJ, H1, K562, and GM12878 human cell lines (Chen *et al.* 2019), which contains $n_{x}=n_{y}=1047$ cells in both modalities. Following (Demetci *et al.* 2022a), we carried out dimensionality reduction on the chromatin accessibility information using the topic modeling framework cisTopic (González-Blas *et al.* 2019), resulting in $p_{x}=19$ dimensions of chromatin accessibility features. We also performed dimensionality reduction on the gene expression information using PCA, resulting in $p_{y}=10$ dimensions of gene expression features.

**Figure 5.**
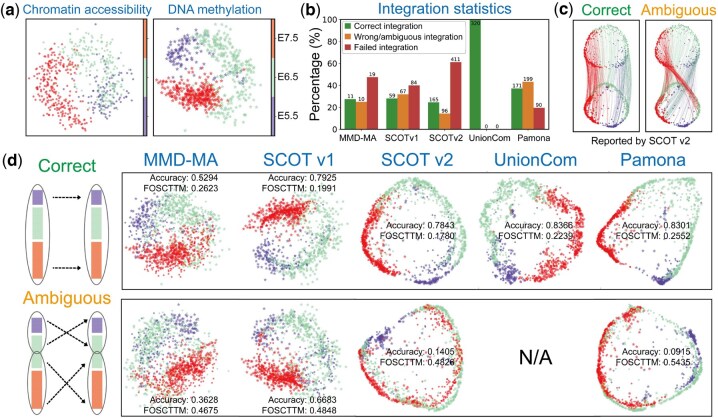
Ambiguous integrations are revealed on the sc-NMT dataset. (a) The dataset consists of two data modalities: chromatin accessibility and DNA methylation. (b) Ambiguous mappings occur across most diagonal integration methods, except for UnionCom, which always succeeds across different settings. It is important to note that ambiguous mappings can only be identified when ground truth labels are available, which are typically absent in real-world data. (c) Ambiguous mappings occur when two parts of one modality are reversely aligned to their counterpart, respectively. (d) Ambiguous mappings can be qualitatively revealed through the aligned manifold and the cross-modality cell–cell correspondences.

The sc-NMT (Fig. 5a) was generated using the sc-NMT sequencing technology (Clark *et al.* 2018), a sequencing technology that simultaneously profiles DNA methylation and chromatin accessibility within the same cell. The dataset was collected from mouse gastrulation at three time points: embryonic day 5.5 (E5.5), E6.5, and E7.5. Following (Cao *et al.* 2020), we filtered out cells where all features were marked as missing values, resulting in $n_{x}=612$ cells in chromatin accessibility and $n_{y}=709$ cells in DNA methylation. A UMAP-based dimensionality reduction (McInnes and Healy 2018) was then performed on each dataset separately, yielding a dimensionality of $p_{x}=p_{y}=300$ for both.

### 2.3 Experimental settings

We investigated the occurrence of artificial integrations caused by ambiguous mapping across various diagonal integration methods. To demonstrate the universality of this issue, we benchmarked several mainstream diagonal integration methods: MMD-MA (Liu *et al.* 2019), SCOT (Demetci *et al.* 2022a, b), UnionCom (Cao *et al.* 2020), and Pamona (Cao *et al.* 2021). We investigated two widely used versions of SCOT: SCOTv1 (Demetci *et al.* 2022a) and SCOTv2 (Demetci *et al.* 2022b), with the latter extending the former by explicitly addressing disproportionate cell types.

We utilized two metrics to quantitatively assess the performance of multimodal single-cell diagonal data integration. Since all simulated and real datasets include cell–cell correspondence information, we used the average “Fraction Of Samples Closer Than the True Match” (FOSCTTM) metric (Liu *et al.* 2019) to quantify how well the integration preserves cell–cell correspondence. Specifically, for each cell in one modality, FOSCTTM calculates the fraction of cells in the other modality that are closer to it than its true match. We then compute the average FOSCTTM by taking the mean of the FOSCTTM values across all cells in both modalities. Since the average FOSCTTM measures the proportion of falsely matched cells, it ranges from 0 to 1, with smaller values indicating better integration performance. Additionally, since both simulated and real datasets contain cell label information, we utilized “Label Transfer Accuracy” (LTA) (Cao *et al.* 2020) to assess how well the shared cell labels can be transferred from one modality to another following the integration. Specifically, LTA evaluates the accuracy of transferring cell labels from one modality to another, based on their neighborhood relationships in the aligned modality. Since LTA measures the proportion of correctly predicted labels, it ranges from 0 to 1, with larger values indicating better integration performance.

## 3 Results

### 3.1 Ambiguous integrations are revealed through simulated data analysis

We first highlighted the artificial integrations arising from ambiguous mapping in simulated datasets (Fig. 2a). We used five mainstream diagonal integration methods to integrate the two modalities of each dataset, utilizing a diverse range of parameter settings recommended by each method. (Refer to S3 for the detailed parameter settings.) Surprisingly, ambiguous mappings occur universally across all these integration methods and are more prevalent than correct mappings (Fig. 2c and Fig. 5, available as supplementary data at *Bioinformatics* online). Thus, blindly trusting the integration results may easily mislead users into pursuing hypotheses based on inaccurate findings.

We then examined the ambiguous mappings both qualitatively and quantitatively. For each dataset, we utilized Principal Component Analysis (PCA) to project the two aligned modalities into a shared 2D space for visualization (Fig. 2b). In the three datasets exhibiting ambiguities, all diagonal integration methods align the branches without grouping those with the same label together. For example, in the T-shaped branch dataset, the green branch in the first modality is incorrectly mapped to the red branch in the second modality, and vice versa. Note that ambiguous mappings can be detected through the cross-modality cell–cell correspondence matrix generated by existing integration methods. In correct integrations, this matrix exhibits a block-diagonal structure, while artificial integrations disrupt this structure by permuting the blocks. Detailed ambiguity analysis results for all five methods, including evaluations using the average FOSCTTM and LTA metrics, are provided in S8.

### 3.2 Artificial integrations are identified through real data analysis

We then highlighted the artificial integrations arising from ambiguous mapping in real datasets. We began by examining the sc-GEM dataset, which comprises two data modalities: chromatin accessibility and DNA methylation (Fig. 3a). Both modalities display similar trajectories with the same order of cell types, transitioning from BJ (human foreskin fibroblast) cells at one end to iPS cells at the other, reflecting the underlying biological process. We used five diagonal integration methods to integrate the two modalities of each dataset with a diverse range of parameter settings (Fig. 3b and Fig. 8, available as supplementary data at *Bioinformatics* online). Surprisingly, ambiguous mappings occur universally across all these integration methods when one modality is reversely aligned to the other during the integration process. Specifically, based on the cell type annotations, iPS cells, located initially at only one end of the trajectory, now reside at both ends of the aligned trajectories (Fig. 3c). The reverse alignment can be revealed qualitatively by the aligned manifold and the cross-modality cell–cell correspondence matrix generated by existing integration methods (Fig. 3d and Fig. 9, available as supplementary data at *Bioinformatics* online).

We next examined the SNARE-Seq dataset, which comprises two data modalities: gene expression and chromatin accessibility (Fig. 4a). Both modalities are derived from a mixture of BJ, H1, K562, and GM12878 human cell lines, with H1, BJ, and K562 cells clustered in different groups. We used five diagonal integration methods to integrate the two modalities of each dataset with a diverse range of parameter settings (Fig. 4b and Fig. 8, available as supplementary data at *Bioinformatics* online). Surprisingly, ambiguous mappings occur across most diagonal integration methods, except for UnionCom, which always fails regardless of parameter settings. It is important to note that ambiguous mappings may not be unique (Fig. 4c and Fig. 10, available as supplementary data at *Bioinformatics* online). For example, one type of ambiguous mappings confuses the correspondence between the BJ cluster and the K562 cluster, while the other type flips the correspondence between the GM cluster and the aggregated BJ/K562 clusters.

Lastly, we examined the sc-NMT dataset, which comprises two data modalities: DNA methylation and chromatin accessibility (Fig. 5a). Both modalities exhibit a similar cyclical trajectory, reflecting the same temporal order of cell collection, transitioning from E5.5 to E6.5 and then to E7.5. We used five diagonal integration methods to integrate the two modalities of each dataset with a diverse range of parameter settings (Fig. 5b and Fig. 8, available as supplementary data at *Bioinformatics* online). Surprisingly, ambiguous mappings occur in most diagonal integration methods, with the exception of UnionCom, which consistently succeeds across various settings. The ambiguous mapping occurs when two segments of one modality (E5.5 and part of E6.5, and E7.5 and part of E6.5) are reversely aligned to their corresponding counterparts (Fig. 5c). The ambiguous mapping can be revealed qualitatively by the cross-modality cell–cell correspondences generated by existing integration methods (Fig. 5d and Fig. 11, available as supplementary data at *Bioinformatics* online). We also quantitatively evaluated the performance of the five diagonal integration methods using the average FOSCTTM and LTA metrics (Fig. 8, available as supplementary data at *Bioinformatics* online).

### 3.3 SONATA consistently diagnoses and improves artificial integrations

**Figure 6.**
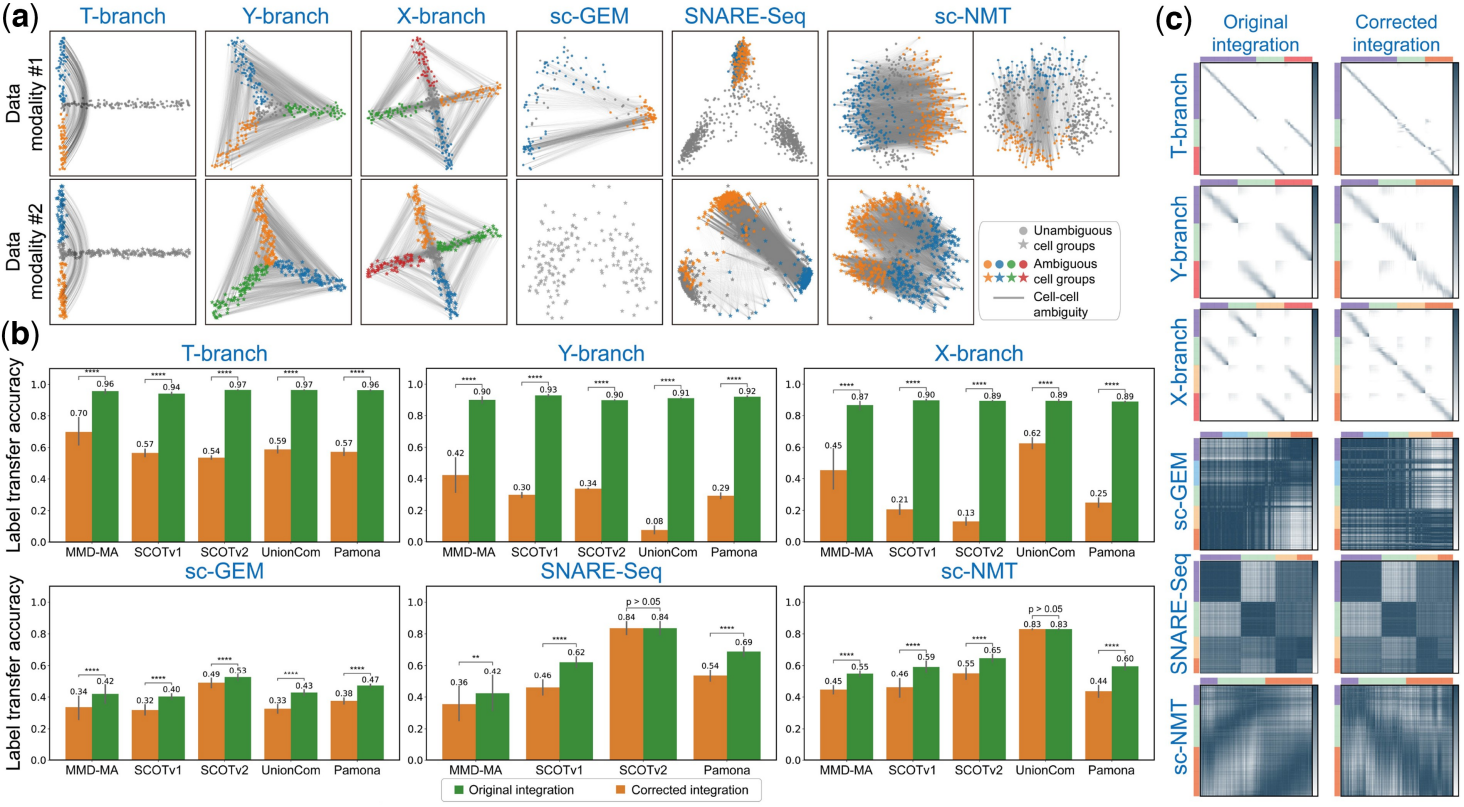
SONATA secures diagonal integrations in both simulated and real datasets. (a) SONATA identifies ambiguous cell groups that may cause confusion during the integration process. (b) SONATA generates alternative integration solutions that are often overlooked by existing methods, among which the best solution demonstrates significantly improved integration performance. For scientific rigor, the performance comparison between the original and the best alternative solution is quantified using one-tailed two-sample *t*-tests to calculate *P*-values: ****P-value≤.0001; ***P-value≤.001; **P-value≤.01; *P-value≤.05. (c) The improved integration performance can be qualitatively observed through the changes in cross-modality cell–cell correspondences.

Given the struggling performance of existing integration methods in both real and simulated datasets, we investigated whether SONATA could assist these methods in identifying ambiguities within the datasets, thereby improving the integration results. As shown in Fig. 6a, SONATA accurately identifies ambiguous cell groups between branches that exhibit geometric resemblance in the simulated datasets. For example, SONATA identifies geometrically similar cell pairs between two and three branches with resemblance in the T-shaped and Y-shaped branch datasets, respectively. The identified ambiguous cell groups are consistent across different modalities, demonstrating SONATA’s robustness in detecting ambiguity regardless of the modality.

In three real datasets, SONATA demonstrates distinct behaviors across different datasets. In the sc-GEM dataset, SONATA identifies the ambiguity between cells at one end of the trajectory (iPS and d24T+ cells) and cells at the opposite end (BJ and d8 cells). The identified ambiguous cell groups are consistent with the observed reverse cross-modality alignment found using five diagonal integration methods (Fig. 3). In the SNARE-Seq dataset, SONATA identifies different ambiguous cell groups across different data modalities. Specifically, in the chromatin accessibility modality, SONATA identifies ambiguous cell groups between the BJ and K562 clusters, while in the gene expression modality, it detects ambiguous groups between the GM cluster and the BJ/K562 clusters. It is important to note that we identified two distinct types of ambiguous mappings while applying five diagonal integration methods for data integration. These mappings correspond precisely to the two ambiguous cell groups reported by SONATA. In the sc-NMT dataset, SONATA identifies different ambiguous cell groups even within a single data modality. Specifically, in the chromatin accessibility modality, SONATA identifies two types of ambiguous cell groups. One type of ambiguous cell group maps E5.5 and a portion of E6.5 to E7.5, while the other type maps E6.5 to sections of both E5.5 and E7.5. Given that the modality follows a cyclical trajectory, E5.5, E6.5, and E7.5 display geometric similarities to one another, making both types of ambiguous cell groups justifiable.

The presence of ambiguous cell groups suggests the existence of alternative yet overlooked integration solutions. Therefore, SONATA leverages these ambiguous cell groups to generate alternative integration solutions that are often overlooked by existing methods. To determine whether SONATA truly uncovers the previously overlooked integrations, we examined whether the alternative solutions lead to improved integration performance quantitatively. As shown in Fig. 6b, the best alternative solution identified by SONATA consistently and significantly outperforms the solutions provided by all existing integration methods. The improved integration performance can be qualitatively observed through the changes in cross-modality cell–cell correspondences (Fig. 6c). The detection of ambiguous cell–cell mappings is interpretable, as statistically confident mappings can be qualitatively visualized for users (Figs 7 and 12, available as supplementary data at *Bioinformatics* online).

Lastly, it is important to note that SONATA cannot, on its own, produce a biologically plausible integration without further investigation. SONATA detects alternative integration solutions often overlooked by existing methods, including biologically plausible integration. Further investigation is needed to identify the biologically plausible integration solution among multiple candidates. For example, as noted by Xu and McCord (2022), prior knowledge can be incorporated–such as a small set of shared features that help reveal cell type identities across modalities, or a limited set of cell anchors derived from joint-profiling technologies at single-cell resolution.

## 4 Discussion and conclusion

In this study, we propose SONATA, a novel diagnostic method for diagonal data integration of multimodal single-cell data that aims to identify potential artificial integrations resulting from ambiguous mapping. The key novelty of SONATA is 2-fold. First and foremost, we demonstrate that artificial integrations resulting from ambiguous mapping in diagonal data integration are widespread yet surprisingly overlooked, occurring across all mainstream diagonal integration methods. Additionally, we define a novel cell–cell ambiguity measurement, assessed through a statistical confidence measure for each pair of cells within the same modality. This ambiguity is used to identify cells with similar geometric contexts, which could potentially lead to artificial integrations in cross-modality mapping.

Unlike existing methods that recklessly report an arbitrary integration solution, SONATA distinguishes whether the integration is unique or if multiple alternative solutions exist, prompting users to carefully discern the true biological solution before proceeding to downstream analysis. Notably, SONATA is not designed to replace any existing pipelines for diagonal data integration; instead, SONATA works simply as an add-on to an existing pipeline for achieving more reliable integration. We applied SONATA to both simulated and real multimodal single-cell datasets to demonstrate its empirical utility. Our experiments on real datasets highlight SONATA’s ability to safeguard diagonal integration of gene expression, DNA methylation, and chromatin accessibility modalities against ambiguous mapping from mainstream methods, effectively informing potential users where these methods might fail.

This study points to several promising directions for future research. SONATA is designed as a post-processing method to complement existing manifold alignment methods. We rationalize that the proposed cell–cell ambiguity measurement can be repurposed to measure the cell–cell similarity between different modalities. Using SONATA directly for both manifold alignment and disambiguation would be interesting directions to pursue. Furthermore, SONATA identifies manifold ambiguity by repeatedly aligning the dataset to its noisy variations. While this iterative self-alignment process enhances robustness, it inevitably introduces computational inefficiency. Exploring more efficient and effective approaches for identifying manifold ambiguity would be an interesting direction for future research. Additionally, several critical single-cell applications (Lan *et al.* 2024a,c,d), such as batch-effect correction (Luecken *et al.* 2022) and comparative analysis between case-control studies (Park and Kellis 2021), are closely associated with the manifold alignment. We would like to use SONATA to identify and resolve the ambiguity in wide applications. Lastly, large language models (LLMs) have been increasingly used to enable researchers without advanced programming skills to easily access sophisticated biomedical data analyses (Dong *et al.* 2023, Lan *et al.* 2024b, 2025). We aim to leverage LLMs to facilitate efficient, professional, and automated integrative data analysis.

## Supplementary Material

btaf345_Supplementary_Data
