# Supplementary material for: Securing diagonal integration of multimodal single-cell data against ambiguous mapping
Source: Bioinformatics. 2025 Jun 14;41(6):btaf345. doi: 10.1093/bioinformatics/btaf345 (PMC12205172; doi:10.1093/bioinformatics/btaf345)
Supplement: btaf345_Supplementary_Data [file btaf345_supplementary_data.pdf]

Supplement to “Securing diagonal integration of multimodal single-cell data against ambiguous mapping”

Han Zhou[1], Kai Cao[2], and Yang Young Lu*[1]

[1]Cheriton School of Computer Science, University of Waterloo, Waterloo, Ontario, Canada
[2]Eric and Wendy Schmidt Center, Broad Institute of MIT and Harvard, Boston, Massachusetts, USA

S1 Related works

Most diagonal integration methods rely on unsupervised manifold alignment [Xu and McCord, 2022], a technique designed to discern a low-dimensional manifold that captures covariation across various data modalities in the absence of cross-modality correspondence information. As one of the pioneering efforts, MATCHER [Welch et al., 2017] employs a Gaussian process latent variable model for integration, albeit constrained to aligning 1-D trajectories. Recent methods, such as MMD-MA [Liu et al., 2019a], SCIM [Stark et al., 2020], SCOT [Demetci et al., 2022a,b], UnionCom [Cao et al., 2020], and Pamona [Cao et al., 2022], have extended the capabilities of MATCHER to accommodate more intricate structures. Among these methods, optimal transport-based approaches such as SCOT and Pamona have gained increasing attention due to their ability to efficiently morph a source population into a target population using only the source and target samples. When cross-modality correspondence information is readily available, diagonal integration simplifies to horizontal or vertical integration [Argelaguet et al., 2021], depending on the presence of shared features or cells, respectively. In horizontal integration, features between modalities exhibit one-to-one correspondence and serve as anchors for integrating the datasets; batch correction methods fall into this category, including Harmony [Korsunsky et al., 2019], Scanorama [Hie et al., 2019], Seurat [Stuart et al., 2019], LIGER [Welch et al., 2019], and so on. For example, Seurat [Stuart et al., 2019] employs canonical correlation analysis to identify shared manifold space, while LIGER [Welch et al., 2019] achieves integration through integrative non-negative matrix factorization. In vertical integration, multiple modalities are concurrently profiled from the same samples, and the datasets are integrated by concatenating the features, including Hetero-RP [Lu et al., 2017], MOFA+ [Argelaguet et al., 2020], iNMF [Liu et al., 2019b], and so on. For example, Hetero-RP [Lu et al., 2017] concatenates and rescales features from different modalities, prioritizing important features with higher weights than others.

S2 Optimal transport

Optimal transport identifies the most cost-effective way to transfer data points from one domain to another. It can be visualized as the problem of moving a pile of sand to fill a hole with the minimal amount of effort. Gromov–Wasserstein optimal transport, a commonly used variant of optimal transport, performs the transport operation by comparing the distances between samples rather than the samples themselves [Alvarez-Melis and Jaakkola, 2018]. Specifically, let two datasets be $X = \{x_i\}_{i=1}^{n_x} \in \mathbb{R}^{n_x \times p_x}$ in data modality $\mathcal{X}$ and $Y = \{y_j\}_{j=1}^{n_y} \in \mathbb{R}^{n_y \times p_y}$ in data modality $\mathcal{Y}$, respectively. The numbers of cells in the two data modalities are n_x and n_y, and the feature dimensions are p_x and p_y, respectively. Next, we construct a weighted k-nearest neighbor (k-NN) graph of cells [Costa and Hero, 2004] and compute the shortest distance between each pair of nodes within each data modality, as these shortest

*Correspondence: yanglu@uwaterloo.ca

distances serve as approximations of geodesic distances on the data manifold [Tenenbaum et al., 2000]. We designate the resultant geodesic matrices for X and Y as $\mathcal{K}_x \in \mathbb{R}^{n_x \times n_x}$ and $\mathcal{K}_y \in \mathbb{R}^{n_y \times n_y}$, respectively.

Given two data modalities and a cost function $L : \mathbb{R} \times \mathbb{R} \to \mathbb{R}$, a fourth-order tensor $\mathbf{L} \in \mathbb{R}^{n_x \times n_x \times n_y \times n_y}$ is computed, where $\mathbf{L}_{ijkl} = L([\mathcal{K}_x]_{ik}, [\mathcal{K}_y]_{jl})$. Intuitively, L quantifies how transporting a pair of samples x_i and x_k in the data modality $\mathcal{X}$ onto another pair of samples y_j and y_l in the data modality $\mathcal{Y}$. Then, the discrete Gromov–Wasserstein problem is defined as:

$$\mathrm{GW}(p_x, p_y) = \min_{\Gamma \in \Pi(p_x, p_y)} \sum_{i,j,k,l} \mathbf{L}_{ijkl} \Gamma_{ij} \Gamma_{kl} \tag{1}$$

where $p_x \in \mathbb{R}^{n_x}$ and $p_y \in \mathbb{R}^{n_y}$ are the marginal distribution in two datasets, respectively. And $\Pi(p_x, p_y)$ is the set of coupling matrices defined as:

$$\Pi(p_x, p_y) = \left\{ \Gamma \in \mathbb{R}_+^{n_x \times n_y} : \Gamma \mathbf{1}_{n_y} = p_x,\ \Gamma^T \mathbf{1}_{n_x} = p_y \right\} \tag{2}$$

One advantage of using optimal transport is its probabilistic interpretation of the resulting coupling matrix Γ, where each entry Γ_{ij} describes how much of the mass of x_i in $\mathcal{X}$ should be mapped to y_j in $\mathcal{Y}$. The summation in Eq. 2 can also be expressed as the inner product $\langle \mathbf{L}(\mathcal{K}_x, \mathcal{K}_y) \otimes \Gamma, \Gamma \rangle$. To improve computational tractability, we solve the entropically regularized version:

$$\mathrm{GW}_\epsilon(p_x, p_y) = \min_{\Gamma \in \Pi(p_x, p_y)} \langle \mathbf{L}(\mathcal{K}_x, \mathcal{K}_y) \otimes \Gamma, \Gamma \rangle - \epsilon H(\Gamma) \tag{3}$$

where $\epsilon > 0$ and $H(\Gamma)$ is the Shannon entropy defined as $H(\Gamma) = \sum_{i=1}^{n_x} \sum_{j=1}^{n_y} \Gamma_{ij} \log \Gamma_{ij}$. Larger values of ϵ make the problem more convex but result in a denser coupling matrix, indicating more correspondences between samples. Conversely, smaller values of ϵ produce sparser solutions, increasing the likelihood of identifying correct one-to-one correspondences between datasets. Eq. 3 can be optimized using a projected gradient descent approach, with both the projection and the gradient computed with respect to the Kullback-Leibler divergence [Peyré et al., 2016].

S3 Baseline settings

To evaluate baseline performance on different datasets, we tested the parameters within the recommended ranges as described in the original publications. For SCOTv1 and SCOTv2, we followed the SCOT tutorial, testing the parameter k between [20, $n/5$], where n denotes the number of samples (cells) in the smallest dataset. Additionally, we followed the tutorial's guidance when testing the range of the coefficient of the entropic regularization term e. For UnionCom, we tested k and ρ based on the robustness analysis outlined in the original work. In the case of MMD-MA, we preprocessed the input data using the linear kernel as described in the literature, and followed the method in [Singh et al., 2020] to automatically compute the bandwidth parameter σ. We experimented with two sets of λ_1 and λ_2 for each dataset, conducting tests across 20 random seeds for each setting.

The detailed parameter settings are provided in Table S1:

S4 Details of SONATA

S4.1 Acceleration of spline fitting

SONATA fits a cubic smoothing spline to model the probability of cell-cell correspondence as a function of geodesic distance. However, fitting a spline directly to all cell-cell pairs may not be ideal due to the computational expense of handling the quadratic number of pairs, and the noise they introduce could negatively impact the fitting performance. To achieve a smooth and efficient spline fit, we follow the intuition that ambiguous cells are unlikely to exist in isolation but are more likely to appear alongside their neighboring cells within the data manifold. Thus, we partition the cells into approximately $\sqrt{n}$ groups using hierarchical clustering, where n is the total number of cells in the dataset. We then use the average correspondence and geodesic distance between the cells in each pair of groups to perform the fit.

S5 Parameter Settings in SONATA

S5.1 Sensitivity analysis of the noise scale

To detect manifold ambiguity in a given data modality, SONATA generates variational versions of the data by introducing random Gaussian noise. In particular, the noise is introduced in a feature-wise fashion. For each feature, we computed the variance across all cells and multiplied it by a noise scaling factor between 0 and 1 to serve as the variance of a zero-mean Gaussian distribution from which the noise was drawn. After introducing random Gaussian noise, we followed the standard diagonal integration procedure by representing the noisy data as a low-dimensional manifold. This was achieved by constructing a weighted k-nearest neighbor graph of the cells, followed by computing a geodesic distance matrix between them. As a result, the self-alignment aligns the two data manifolds derived from the noisy data, respectively.

The noisy data is crucial for detecting manifold ambiguity via self-alignment. To evaluate how the noise scale influences manifold ambiguity detection through cell–cell correspondences, we assessed the robustness of the detected ambiguous cell pairs across different noise scaling factors between 0 and 1. As shown in Fig. S1**a**, on the real SNARE-seq dataset, the detected ambiguous cell pairs remain similar–as measured by Jaccard similarity–across a range of noise scaling factors, provided the scale is neither too small nor too large. For example, in the chromatin accessibility modality, ambiguity is not detected at very small noise scales (*e.g.,* 0.1), suggesting insufficient perturbation, whereas excessively large noise (*e.g.,* ≥ 0.8) results in unstable and inconsistent outcomes. This observation is reasonable: on one hand, negligible noise can lead to a trivial solution in which each cell aligns with itself rather than with geometrically similar but distant cells on the data manifold. On the other hand, excessive noise can distort the underlying manifold structure, compromising alignment quality and obscuring genuine relationships within the data.

Although SONATA is robust across a wide range of noise scales, it is desirable to provide an empirical guideline for practitioners to select an appropriate noise scaling factor. In principle, the selection of the noise scaling factor should be **data-dependent**, as different manifolds exhibit varying sensitivity to perturbations. For example, a data modality with a clear and low-noise structure remains robust even under a large noise scale without losing manifold coherence, whereas a modality already affected by substantial noise is more susceptible to further disruption. To guide the selection of the noise scaling factor, we offer a data-driven strategy grounded in the manifold similarity criterion. Specifically, we compute the Pearson correlation between the geodesic distance matrices of the original and noisy data. This metric quantifies the extent to which the original manifold is preserved under different noise scales. As shown in Fig. S1**b**, we observe that maintaining Pearson correlation between 80% and 90% with the original manifold yields robust results across datasets, striking a balance between introducing meaningful manifold variation and preserving manifold fidelity.

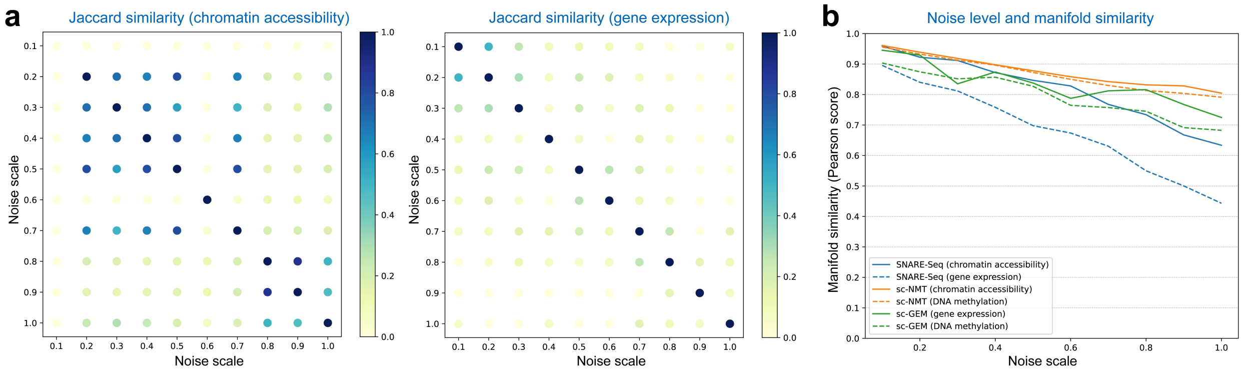

Figure S1: **The sensitivity analysis of the noise scale.** (**a**) SONATA is robust across a wide range of noise scales on the real SNARE-seq dataset, provided the scale is neither too small nor too large. The consistency of detected ambiguous cell pairs between two different noise scaling factors is measured using the Jaccard similarity. (**b**) A data-driven strategy is provided to guide the selection of the noise scaling factor based on the manifold similarity criterion.

S5.2 Sensitivity analysis of the number of neighbors

To detect manifold ambiguity in a given data modality, SONATA performs a self-alignment procedure that aligns the two data manifolds derived from the Gaussian-based and neighbor-based noisy data, respectively. In the latter, a modified k-nearest neighbor (k-NN) graph is constructed by randomly adding k second-order neighbors for each cell. Geodesic distances are then approximated using shortest paths on the graph.

To evaluate how the number of neighbors affects manifold ambiguity detection via cell–cell correspondences, we assessed the robustness of the detected ambiguous cell pairs across varying values of k. As shown in Fig. S2, on the real SNARE-seq dataset, the detected ambiguous cell pairs remain similar–as measured by Jaccard similarity–across a wide range of k. Thus, we conclude that SONATA is robust to the choice of the number of neighbors used in the OT-based self-alignment for revealing manifold ambiguity.

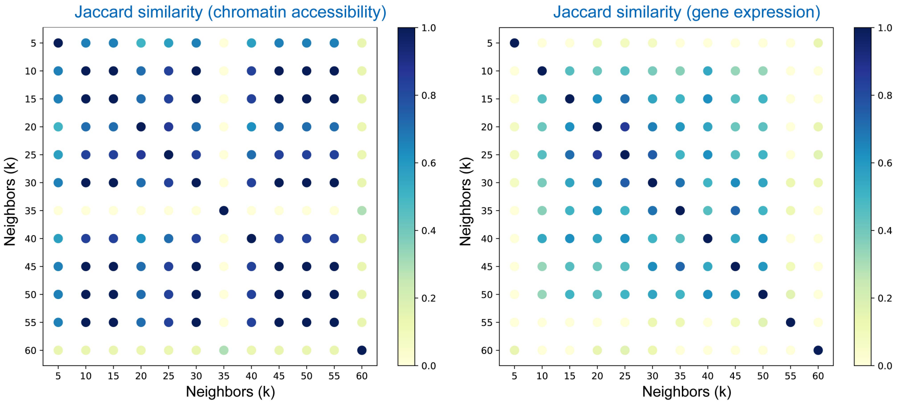

Figure S2: **The sensitivity analysis of the number of neighbors.** SONATA is robust across a wide range of number of neighbors on the real SNARE-seq dataset. The consistency of detected ambiguous cell pairs between two different noise scaling factors is measured using the Jaccard similarity.

S6 Scalability analysis

We assessed computational scalability by breaking down SONATA's runtime into its components and analyzing how each scales with the number of cells. Specifically, we divided the overall process into two stages: (1) OT-based self-alignment to reveal manifold ambiguity, and (2) detection of ambiguous groups based on the self-alignment results. To evaluate scalability, we measured SONATA's running time on simulated T-branch datasets with increasing numbers of cells. As shown in Fig. S3**a**, the majority of the computational cost stems from the OT-based self-alignment. As SONATA performs multiple rounds of variational self-alignment and optimal transport calculations, it can incur substantial computational overhead, potentially limiting its scalability to large-scale datasets.

To overcome the computational bottleneck of OT-based self-alignment, we adopt a more efficient yet equally powerful optimal transport algorithm known as Quantized Gromov–Wasserstein or scalable OT [Chowdhury et al., 2021]. This method enhances scalability by dividing the measures into smaller partitions, matching them based on representative points, and recursively solving OT within each matched pair. As shown in Fig. S3**b**, compared to standard OT, whose runtime grows rapidly with the number of cells, scalable OT exhibits relatively flat runtime scaling, demonstrating substantial improvements in computational efficiency as the dataset size increases.

Despite the efficiency gains from the scalable OT, it is important to note that scaling SONATA to extremely large datasets involves additional factors. As shown in Fig. S3**c**, despite its computational efficiency, the peak memory usage of scalable OT remains comparable to that of standard OT (Fig. S3**c**). In addition, ambiguous group detection is formulated as a constrained clustering problem [Basu et al., 2004], where pairwise ambiguity constraints are incorporated to ensure that any two cells identified as ambiguous are not assigned to the same group. It is worth mentioning that SONATA employs an open-source constrained clustering implementation, Active Semi-Supervised Clustering (`https://github.com/datamole-ai/active-semi-supervised-clustering`), which becomes less efficient when applied to extremely large datasets. Therefore, accelerating the constrained clustering

algorithm or exploring alternative scalable and memory-efficient methods for detecting ambiguous cell groups are promising directions for future work.

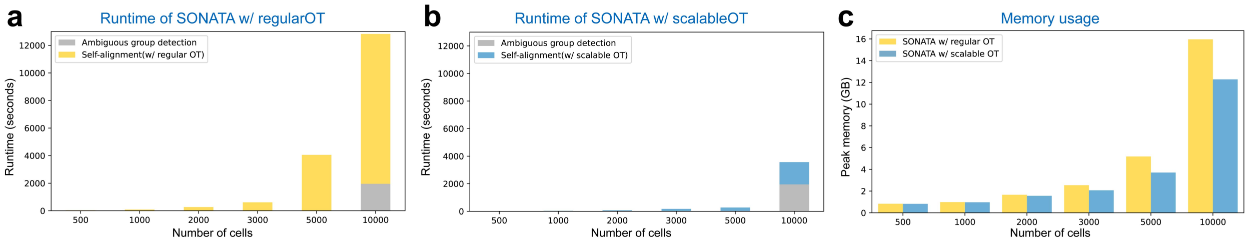

Figure S3: **The scalability analysis of SONATA.** SONATA's runtime is divided into two stages: OT-based self-alignment and ambiguous group detection. We compare two OT algorithms: (**a**) standard OT and (**b**) scalable OT. For each setting, self-alignment is repeated ten times, and the results are aggregated to ensure robustness. (**c**) Despite its computational efficiency, the peak memory usage of scalable OT remains comparable to that of standard OT. For a fair comparison, all methods were executed in the same computational environment: a dual-socket AMD EPYC 7302 16-core processor and 32 GB of DDR4 RAM.

S7 Alternative visualization

For each dataset, we utilized Principal Component Analysis (PCA) to project the data modalities into a 2D space for visualization. While PCA is a commonly used dimensionality reduction method to illustrate manifold ambiguity, it is important to assess the robustness and consistency of the detected ambiguity under alternative dimensionality reduction techniques. To this end, we employed two alternative yet widely used dimensionality reduction methods: t-SNE [der Maaten and Hinton, 2008] and UMAP [McInnes and Healy, 2018]. As shown in Fig. S4, on the real SNARE-seq dataset, projecting the data modalities (*e.g.,* chromatin accessibility) into a 2D space using t-SNE and UMAP reveals ambiguity patterns similar to those observed with PCA. This demonstrates that the visual interpretation of the results remains stable across different visualization techniques.

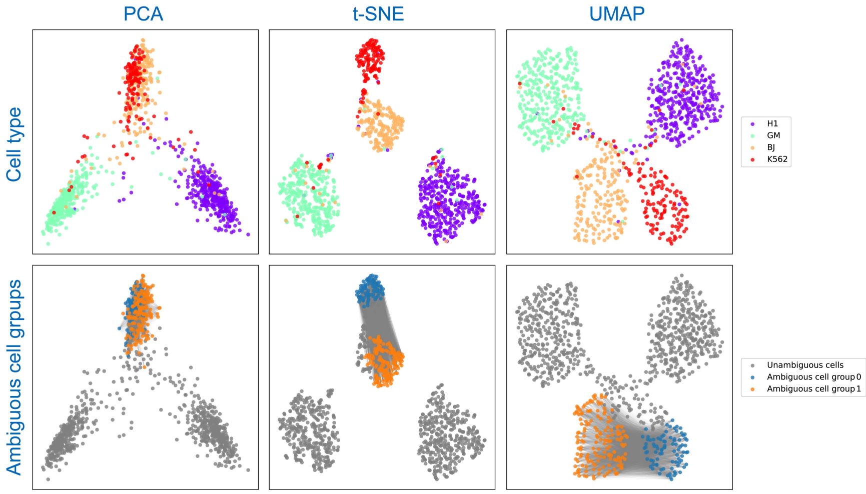

Figure S4: **Different visualization of the SNARE-seq dataset.**

S8 Simulated data analysis

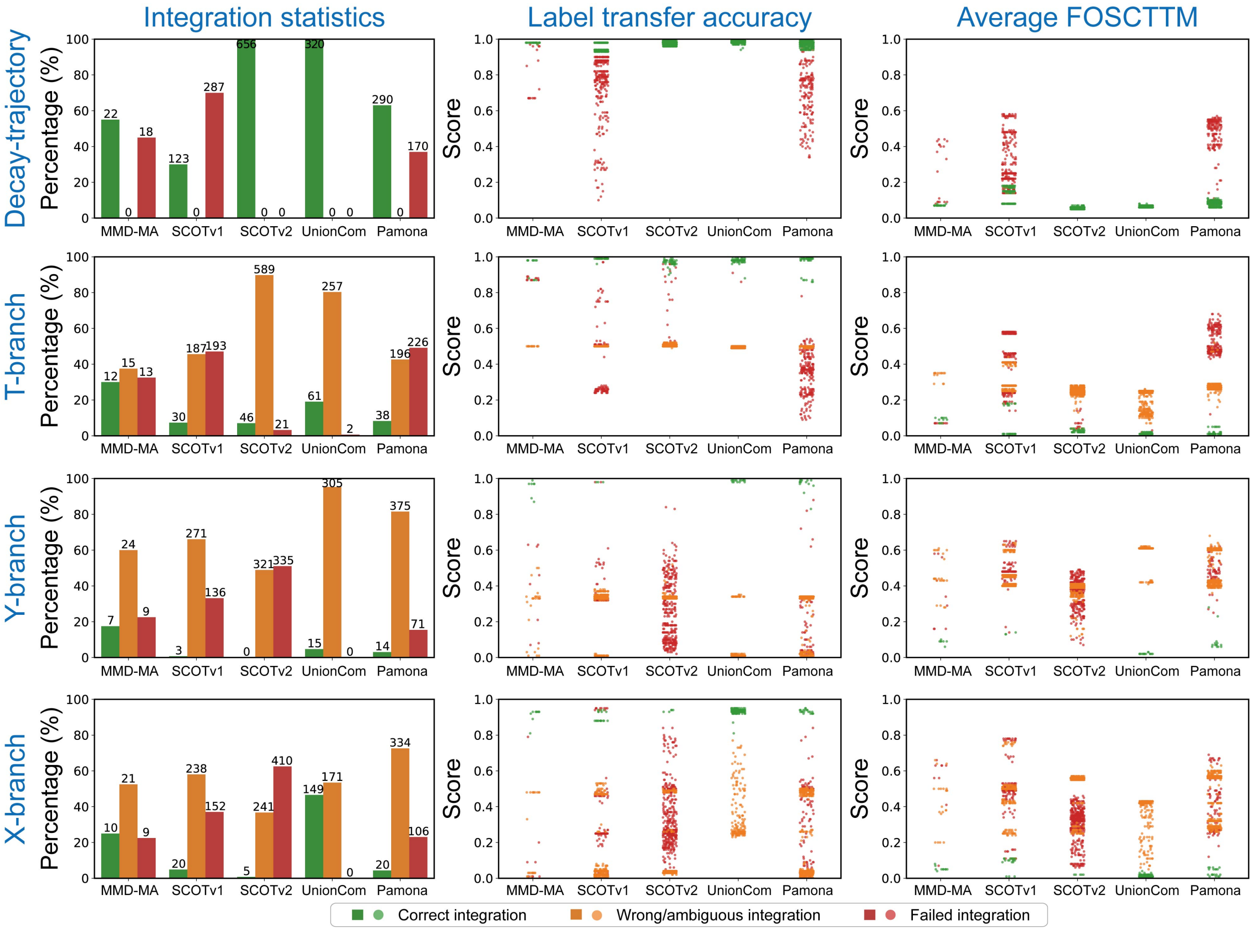

Figure S5: **Ambiguous mappings occur universally across mainstream diagonal integration methods on simulated datasets.** The diagonal integration performance is quantitatively measured by LTA and FOSCTTM.

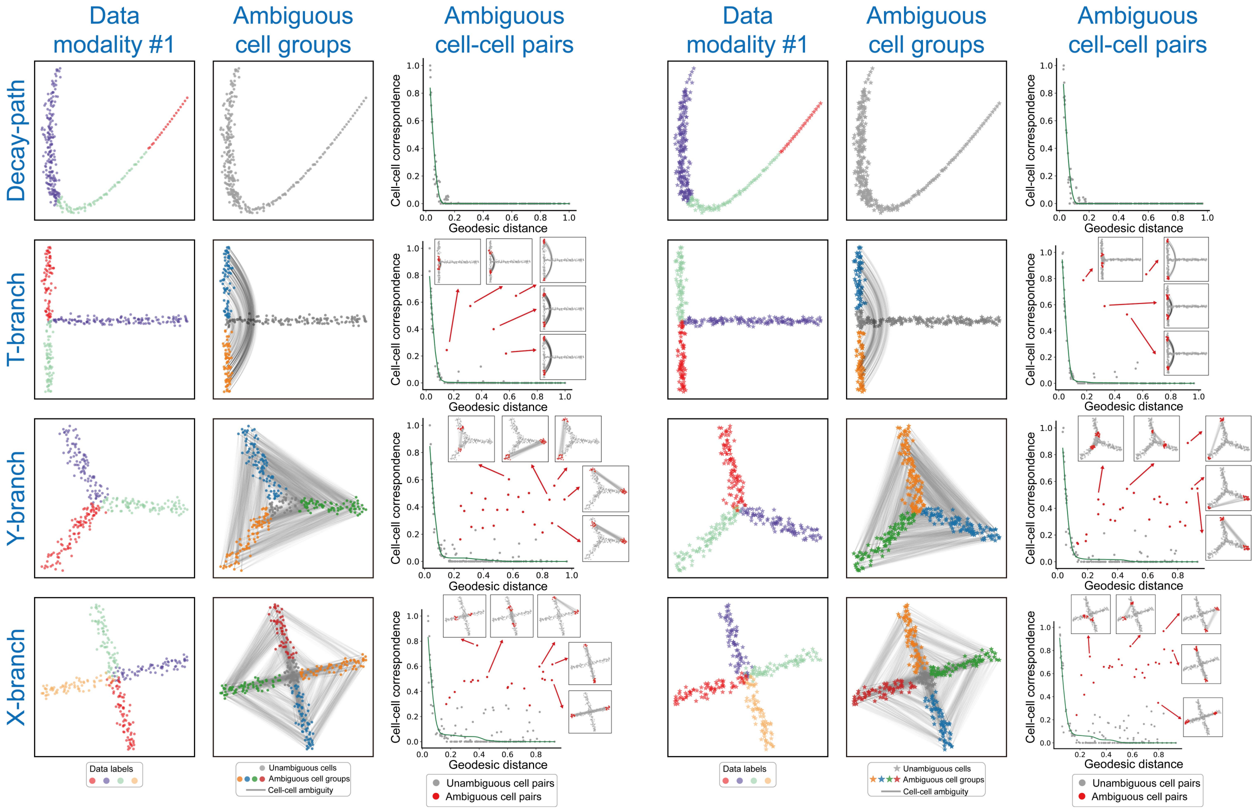

Figure S6: **Comprehensive SONATA results on simulated datasets.**

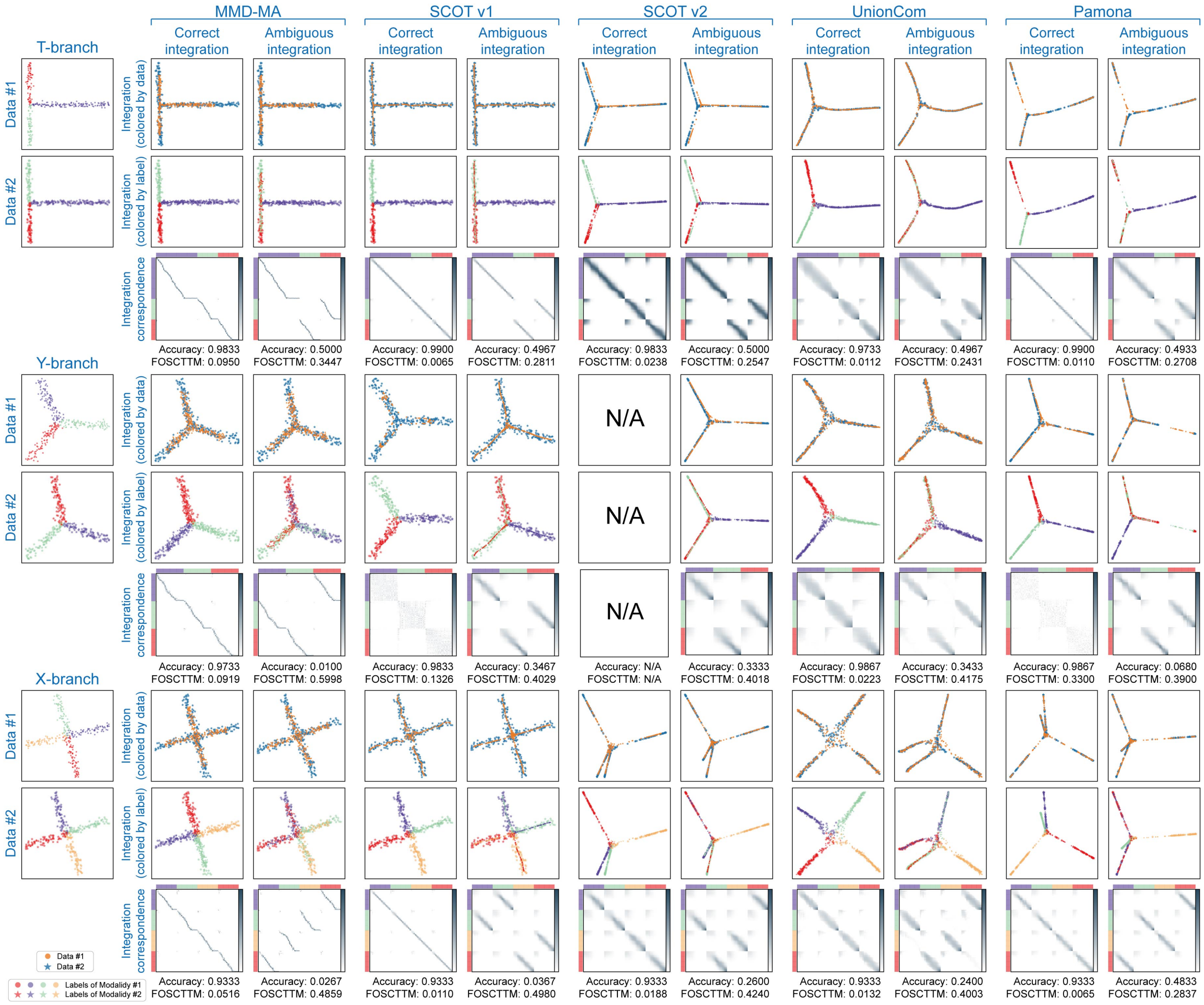

Figure S7: **Comprehensive ambiguous integration results on simulated datasets.**

S9 Real data analysis

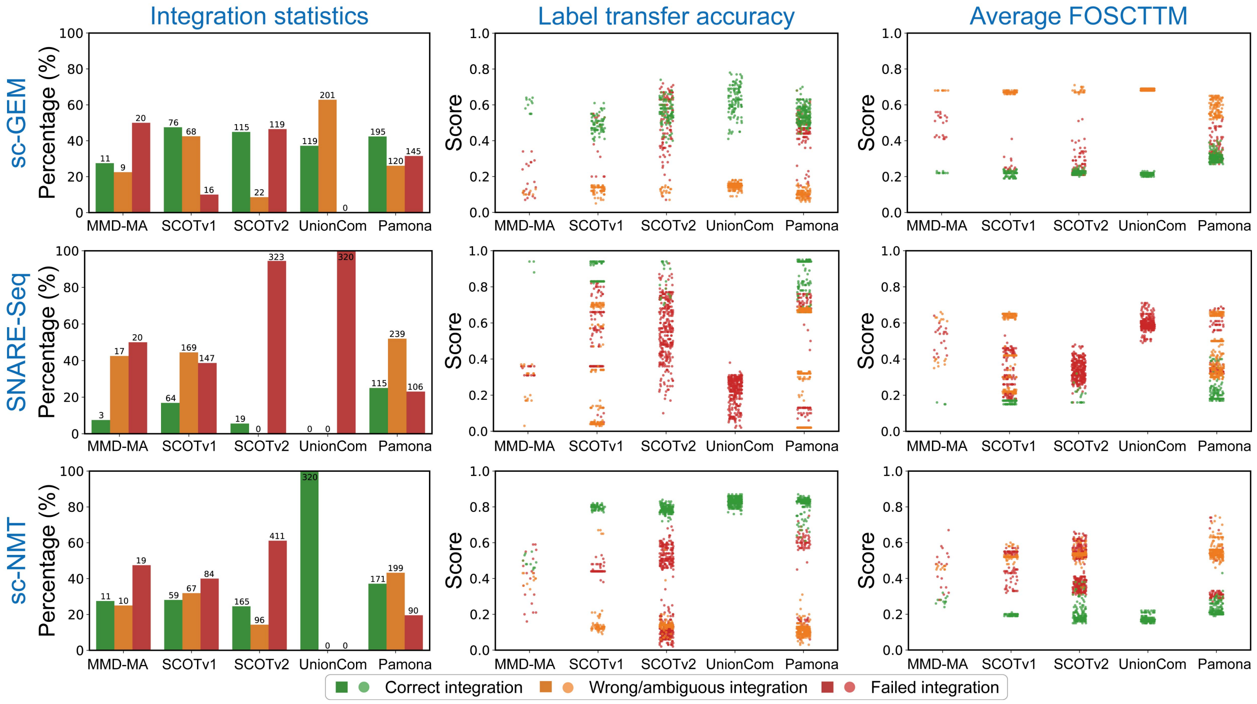

Figure S8: **Ambiguous mappings occur universally across mainstream diagonal integration methods on three real datasets.** The diagonal integration performance is quantitatively measured by LTA and FOSCTTM. It is important to note that ambiguous mappings can only be identified when ground truth labels are available, which are typically absent in real data.

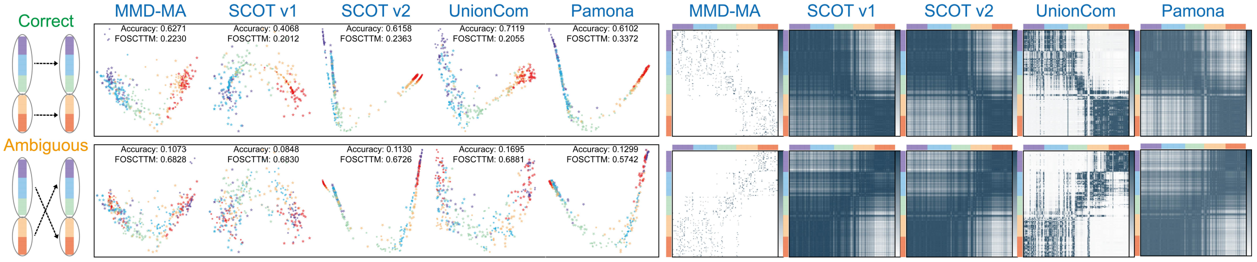

Figure S9: **Ambiguous mappings on the sc-GEM dataset.** Ambiguous mappings can be qualitatively revealed through the aligned manifold and the cross-modality cell-cell correspondences.

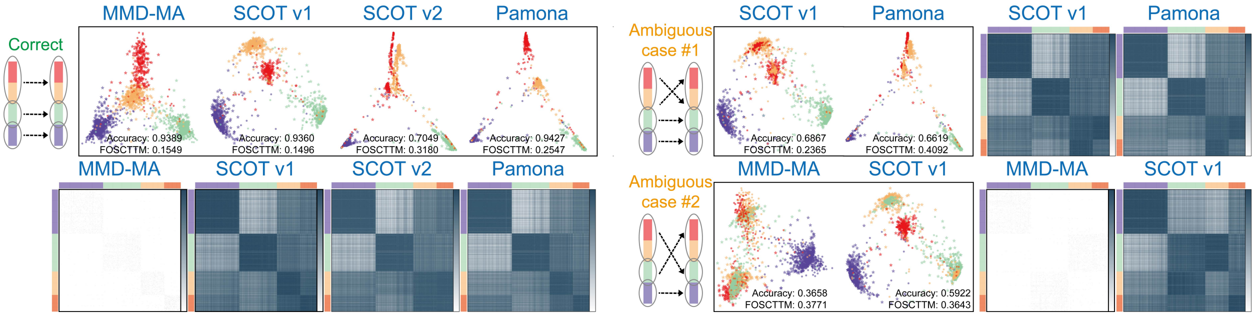

Figure S10: **Ambiguous mappings on the SNARE-Seq dataset.** Ambiguous mappings can be qualitatively revealed through the aligned manifold and the cross-modality cell-cell correspondences. Artificial integrations can result from different types of ambiguous mappings.

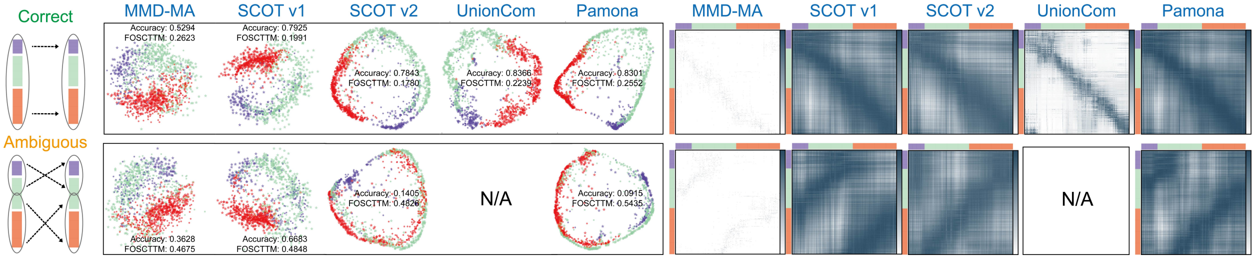

Figure S11: **Ambiguous mappings on the sc-NMT dataset.** Ambiguous mappings can be qualitatively revealed through the aligned manifold and the cross-modality cell-cell correspondences.

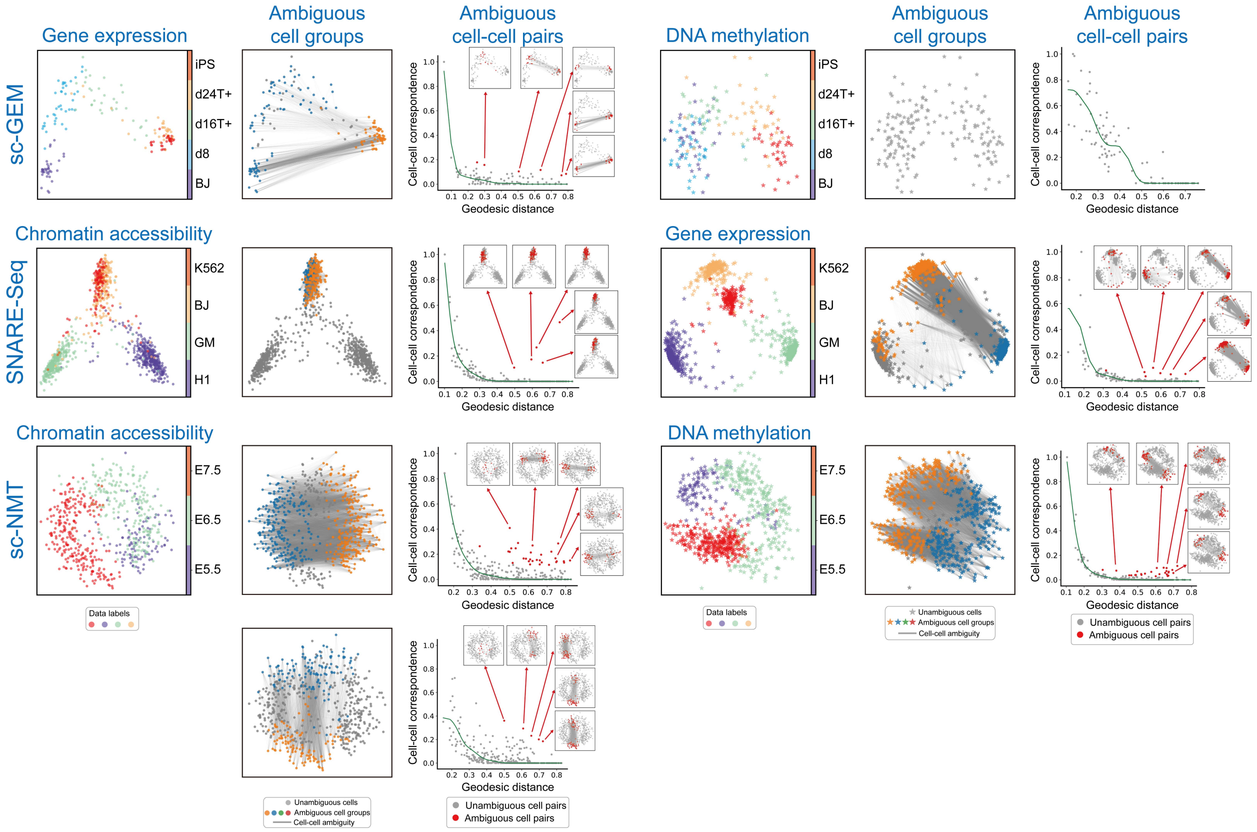

Figure S12: **Comprehensive SONATA results on real datasets.**

References

D. Alvarez-Melis and T. S. Jaakkola. Gromov-wasserstein alignment of word embedding spaces. arXiv preprint arXiv:1809.00013, 2018.

R. Argelaguet, D. Arnol, D. Bredikhin, Y. Deloro, B. Velten, J. C. Marioni, and O. Stegle. MOFA+: a probabilistic framework for comprehensive integration of structured single-cell data. Genome Biology, 21(111), 2020.

R. Argelaguet, A. S. Cuomo, O. Stegle, and J. C. Marioni. Computational principles and challenges in single-cell data integration. Nature Biotechnology, 39(10):1202–1215, 2021.

S. Basu, A. Banerjee, and R. J. Mooney. Active semi-supervision for pairwise constrained clustering. In IEEE International Conference on Data Mining (ICDM), pages 333–344. SIAM, 2004.

K. Cao, X. Bai, Y. Hong, and L. Wan. Unsupervised topological alignment for single-cell multi-omics integration. Bioinformatics, 36(Supplement_1):i48–i56, 2020.

K. Cao, Y. Hong, and L. Wan. Manifold alignment for heterogeneous single-cell multi-omics data integration using Pamona. Bioinformatics, 38(1):211–219, 2022.

S. Chowdhury, D. Miller, and T. Needham. Quantized gromov-wasserstein. In Machine Learning and Knowledge Discovery in Databases. Research Track: European Conference, ECML PKDD 2021, Bilbao, Spain, September 13–17, 2021, Proceedings, Part III 21, pages 811–827. Springer, 2021.

J. A. Costa and A. O. Hero. Manifold learning using euclidean k-nearest neighbor graphs [image processing examples]. In 2004 IEEE International Conference on Acoustics, Speech, and Signal Processing, volume 3, pages iii–988. IEEE, 2004.

P. Demetci, R. Santorella, B. Sandstede, W. S. Noble, and R. Singh. SCOT: Single-cell multi-omics alignment with optimal transport. Journal of Computational Biology, 29(1), 2022a.

P. Demetci, R. Santorella, B. Sandstede, and R. Singh. Unsupervised integration of single-cell multi-omics datasets with disproportionate cell-type representation. In International Conference on Research in Computational Molecular Biology, pages 3–19. Springer, 2022b.

L. V. der Maaten and G. Hinton. Visualizing data using t-SNE. Journal of Machine Learning Research, 9(2579-2605): 85, 2008.

B. Hie, B. Bryson, and B. Berger. Efficient integration of heterogeneous single-cell transcriptomes using scanorama. Nature Biotechnology, 37(6):685–691, 2019.

I. Korsunsky, N. Millard, J. Fan, K. Slowikowski, F. Zhang, K. Wei, Y. Baglaenko, M. Brenner, P. Loh, and S. Raychaudhuri. Fast, sensitive and accurate integration of single-cell data with Harmony. Nature Methods, 16:1289–1296, 2019.

J. Liu, Y. Huang, R. Singh, J.-P. Vert, and W. S. Noble. Jointly embedding multiple single-cell omics measurements. In International Workshop on Algorithms in Bioinformatics, volume 143, pages 10:1–10:13, 2019a.

L. Liu, C. Liu, A. Quintero, L. Wu, Y. Yuan, M. Wang, M. Cheng, L. Leng, L. Xu, G. Dong, et al. Deconvolution of single-cell multi-omics layers reveals regulatory heterogeneity. Nature Communications, 10(1):470, 2019b.

Y. Y. Lu, J. Lv, J. A. Fuhrman, and F. Sun. Towards enhanced and interpretable clustering/classification in integrative genomics. Nucleic Acids Research, 45(20):e169–e169, 2017.

L. McInnes and J. Healy. UMAP: Uniform manifold approximation and projection for dimension reduction. arXiv, 2018.

G. Peyré, M. Cuturi, and J. Solomon. Gromov–wasserstein averaging of kernel and distance matrices. In International Conference on Machine Learning, pages 2664–2672. PMLR, 2016.

R. Singh, P. Demetci, G. Bonora, V. Ramani, C. Lee, H. Fang, Z. Duan, X. Deng, J. Shendure, C. Disteche, et al. Unsupervised manifold alignment for single-cell multi-omics data. In International Conference on Bioinformatics, Computational Biology and Health Informatics, pages 1–10, 2020.

S. G. Stark, J. Ficek, K. Lehmann, X. Bonilla, F. Locatello, G. Rätsch, S. Chevrier, and F. Singer. SCIM: Universal single-cell matching with unpaired feature sets. Bioinformatics, 36(Supplement_2):i919–i927, 2020.

T. Stuart, A. Butler, P. Hoffman, C. Hafemeister, E. Papelexi, W. M. M. III, Y. Hao, M. Stoeckius, P. Smibert, and R. Satija. Comprehensive integration of single-cell data. Cell, 77(7):1888–1902, 2019.

J. B. Tenenbaum, V. de Silva, and J. C. Langford. A global geometric framework for nonlinear dimensionality reduction. Science, 290:2319–2323, 2000.

J. D. Welch, A. J. Hartemink, and J. F. Prins. MATCHER: manifold alignment reveals correspondence between single cell transcriptome and epigenome dynamics. Genome Biology, 18(1):138, 2017.

J. D. Welch, V. Kozareva, A. Ferreira, C. Vanderburg, C. Martin, and E. Z. Macosko. Single-cell multi-omic integration compares and contrasts features of brain cell identity. Cell, 177(7):1873–1887, 2019.

Y. Xu and R. P. McCord. Diagonal integration of multimodal single-cell data: potential pitfalls and paths forward. Nature Communications, 13(1):3505, 2022.

Table S1: Hyperparameter settings for baselines.

Method	Fixed Parameters	Tested Parameters
MMD-MA	$num\_feat = 4$, $epoch = 5000$	$\lambda_1, \lambda_2 \in [(1e-3, 1e-3), (1e-6, 1e-7)]$ for Decay-trajectory<br>$\lambda_1, \lambda_2 \in [(1e-3, 1e-3), (1e-6, 1e-6)]$ for other simulated datasets<br>$\lambda_1, \lambda_2 \in [(1e-3, 1e-3), (1e-6, 1e-6)]$ for sc-GEM<br>$\lambda_1, \lambda_2 \in [(1e-3, 1e-3), (1e-5, 1e-5)]$ for SNARE-Seq<br>$\lambda_1, \lambda_2 \in [(1e-3, 1e-3), (1e-5, 1e-6)]$ for sc-NMT<br>$random\_seed \in [0, 1, 2, \ldots, 19]$ for all datasets
SCOT v1	Default Settings	$k \in [20, 21, 22, \ldots, 60]$ for simulated datasets<br>$k \in [20, 21, 22, \ldots, 35]$ for sc-GEM<br>$k \in [20, 25, 30, \ldots, 205]$ for SNARE-Seq<br>$k \in [20, 25, 30, \ldots, 120]$ for sc-NMT<br>$e \in [1e-3, 5e-3, 1e-2, 5e-2, 1e-1]$ for all datasets<br>Tested Settings:<br>1. `mode=distance, metric=euclidean`, for all datasets<br>2. `mode=connectivity, metric=correlation` for all datasets
SCOT v2	Default Settings	$k \in [20, 21, 22, \ldots, 60]$ for simulated datasets<br>$k \in [20, 21, 22, \ldots, 35]$ for sc-GEM<br>$k \in [20, 25, 30, \ldots, 205]$ for SNARE-Seq<br>$k \in [20, 25, 30, \ldots, 120]$ for sc-NMT<br>$e \in [1e-2, 5e-2, 1e-1]$ for SNARE-Seq<br>$e \in [5e-3, 1e-2, 5e-2, 1e-1]$ for simulated, sc-GEM and sc-NMT<br>$\rho \in [1e-3, 1e-2, 1e-1, 1.0]$ for all datasets<br>Tested Settings:<br>1. `mode=distance, metric=euclidean` for all datasets,<br>2. `mode=connectivity, metric=correlation` for sc-NMT
UnionCom	Default Settings	$k \in [5, 6, 7, \ldots, 20]$ for all datasets<br>$\rho \in [1, 2, 3, \ldots, 20]$ for all datasets
Pamona	Default Settings	$k \in [5, 6, 7, \ldots, 50]$ for all datasets<br>$e \in [1e-3, 5e-3, 1e-2, 5e-2, 1e-1]$ for all datasets<br>Tested Settings:<br>1. `mode=distance, metric=minkowski` for all datasets,<br>2. `mode=connectivity, metric=correlation` for all datasets
